# Information can explain the dynamics of group order in animal collective behaviour

**DOI:** 10.1038/s41467-020-16578-x

**Published:** 2020-06-01

**Authors:** Hannah E. A. MacGregor, James E. Herbert-Read, Christos C. Ioannou

**Affiliations:** 1 School of Biological Sciences, University of Bristol, Bristol, BS8 ITQ UK; 2 Department of Zoology, University of Cambridge, Cambridge, CB2 3EJ UK; 3 Department of Biology, Aquatic Ecology Unit, Lund University, Lund, 223 62 Sweden

**Keywords:** Behavioural ecology, Animal behaviour

## Abstract

Animal groups vary in their collective order (or state), forming disordered swarms to highly polarized groups. One explanation for this variation is that individuals face differential benefits or costs depending on the group’s order, but empirical evidence for this is lacking. Here we show that in three-spined sticklebacks (*Gasterosteus aculeatus*), fish that are first to respond to an ephemeral food source do so faster when shoals are in a disordered, swarm-like state. This is because individuals’ visual fields collectively cover more of their environment, meaning private information is more readily available in disordered groups. Once social information becomes available, however, the arrival times of subsequent group members to the food are faster in more ordered, polarized groups. Our data further suggest that first responding individuals (those that benefit from group disorder) maintain larger differences in heading angle to their nearest neighbours when shoaling, thereby explaining how conflict over whether private or social information is favoured can drive dynamic changes in collective behaviour.

## Abstract (web summary)

In animal groups, the degree of alignment of individuals could have different benefits and costs for individuals depending on their reliance on private or social information. Here the authors show that in shoals of three-spined sticklebacks, some individuals reach resources faster when groups are disordered, a state which favours reliance on privately acquired information, while other individuals reach resources faster when groups are ordered, allowing them to exploit social information more effectively.

## Introduction

One of the most conspicuous characteristics of moving animal groups is their degree of spatial and directional organization. At opposite extremes, groups can form disordered states with low directional alignment or highly ordered states where individuals move in the same direction^1–4^. Even within the same group, collective order often fluctuates dynamically over short periods of time and individual-based models of collective movement have highlighted that small, local adjustments to behaviour are responsible for this variation^1,3,5^.

Despite progress in understanding the mechanics of how different group structures arise, why animal groups might adopt different levels of organization over time is poorly understood. Collective order can change due to noise in individuals’ social interactions^6^, suggesting that in some cases, there may be no adaptive benefits to individuals of different group orders^7^. Alternatively, different group structures may offer different advantages related to information acquisition, foraging or avoiding predation^8–10^, with group structure changing adaptively in response to individuals’ hunger, food availability or predation risk. For example, when behaviours evolve to heighten the sensitivity of individuals in groups to unpredictable information, transitions in collective order are predicted to occur more frequently^5,11^.

Within-group conflict has received limited attention as a functional explanation for dynamic fluctuations in collective order, despite conflict between individuals being widespread in animal social groups^12^. If certain individuals perform disproportionately better or worse as a function of the groups’ collective order, rather than being a mutually beneficial outcome as has been suggested previously, fluctuations in group structure and organization could instead reflect inherent conflicts of interest between group members. Recent studies have highlighted how individuals have differing goals, strategies, motivations and experience^13^, and may also rely differently on private and social information^14^. Hence, it is plausible that transitions in order could be driven by divergent individual preferences for the group’s organization. These preferences may vary over time and contexts as individuals attempt to balance important fitness-related trade-offs^15^. Establishing whether individuals benefit from different collective orders is challenging, as this requires detailed spatiotemporal data on the behaviour of individuals within groups that are performing ecologically relevant and functionally important tasks.

Here we investigate the role of individual and group-level properties, including collective order, in the foraging performance of three-spined sticklebacks (*Gasterosteus aculeatus*). Groups of individuals were tasked with detecting and reaching a standardized visual stimulus that mimicked an ephemeral food source. Faster response times during scramble competition contribute to greater food consumption, access to preferred food items and avoidance of competition; therefore, the speed at which resources are reached will have important fitness consequences for individuals. Sticklebacks form shoals outside of the breeding season and are a model species to use in experiments of scramble competition, because individuals will compete for resources when they simultaneously encounter small food patches or single prey items such as drifting invertebrates^16,17^. As individuals can use privately or socially (from behavioural cues) acquired information to detect resources^18^, we tested whether the speed that individuals responded to and reached the resource was different when groups adopted different collective orders. Although our focus is on food resources, many of our results should also apply to the detection by groups of other hard-to-detect stimuli, such as ambush predators^19,20^.

Shoals of eight fish were allowed to freely explore an arena^2^ and their behavioural responses to the appearance of a red-tipped pipette that delivered a food item unpredictably at one of four locations in the arena were tracked at a high spatial and temporal resolution^21^ (Supplementary Fig. 1 and Supplementary Movie 1). A minimum distance (43 cm) between the fish and the stimulus at the time of presentation increased the challenge of detecting the stimulus for the fish, mimicking group foraging for ephemeral resources in the natural environment. To test the effects of individual-level behaviour on the likelihood of being the first fish to respond, we calculated six parameters from the trajectory data: (i) swimming speed; (ii) distance to the stimulus; (iii) bearing to the stimulus, the degree of orientation of the fish towards the stimulus; (iv) proportion of time on the convex hull edge of the group, a measure of the tendency of the fish to be towards the centre or on the edge of the group^22^; (v) distance to the group centroid; and (vi) visual occlusion^23^, an estimate of visual interference by near neighbours. To examine the relationships between group behaviour and the latency of the first individual to respond to the stimulus, and separately the latency to arrive at the stimulus for each individual, we calculated five parameters: (i) convex hull area, a measure of group cohesion; (ii) bearing of the group heading to the stimulus, the degree of orientation of the group towards the stimulus; (iii) distance of the group centroid to the stimulus; (iv) centroid speed; and (v) polarization^1,2^, a measure of collective order between 0 (no directional alignment between individuals) and 1 (all individuals are perfectly aligned, see Supplementary Methods for details of how all parameters were calculated).

## Results

**Fig. 1:**
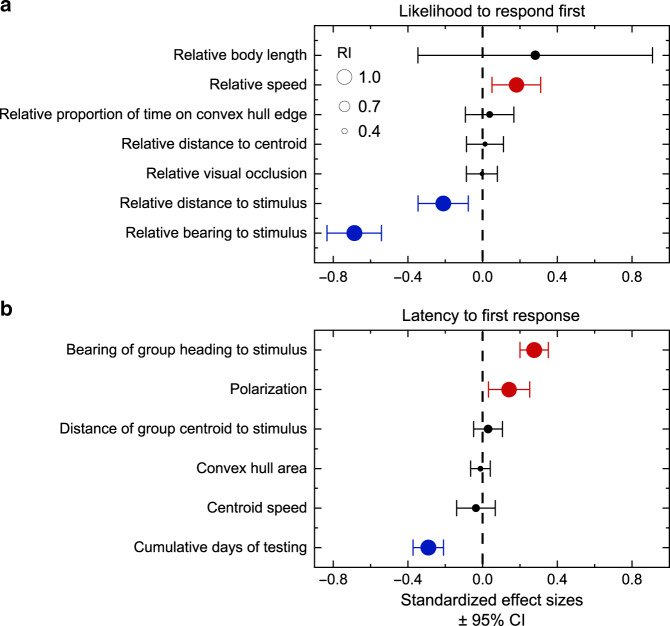
Predictors of responsiveness to the stimulus. Model averaged coefficient estimates from candidate generalized linear mixed models (GLMMs) to examine the predictors of (**a**) the likelihood of first response and (**b**) the latency to first respond (*n* = 428 presentations). Centres of points indicate the standardized model averaged effect sizes for each of the parameters (scaled, mean = 0, SD = 1) and error bars indicate 95% confidence intervals (CI) of effect sizes based on the 95% candidate set of models (Supplementary Tables 2 and 4). Sizes of points represent the relative importance (RI) of each parameter: the sum of the Akaike weights over the models in which that parameter appears (based on the complete candidate set of models). Parameters that co-vary positively with the response variable are coloured red, whereas those that co-vary negatively are coloured blue.

We first used a model comparison approach to assess what factors influenced the likelihood that an individual was the first to respond to the stimulus (Supplementary Fig. 2, see Methods). Although previous studies have emphasized that an individual’s spatial position within the group can determine its likelihood of response^23,24^, only parameters that did not depend on the position or orientation of other individuals in the group were important predictors of the likelihood that an individual responded first (Fig. 1a and Supplementary Tables 1 and 2). Fish that were closer to the stimulus (relative importance (RI) = 0.98) and orientated towards (i.e., facing) the stimulus (RI = 1.00), relative to other individuals in the shoal, were more likely to respond first (Fig. 1a and Supplementary Fig. 3a, b). Individuals’ relative speed (RI = 0.96) and relative body length (RI = 0.59) were also positive predictors of their likelihood to respond first (Fig. 1a and Supplementary Fig. 3c, d). The proportion of time spent on the convex hull edge of the group, distance to the group centroid and visual occlusion relative to other individuals in the shoal were not strong predictors of the likelihood to respond first to the stimulus (Fig. 1a and Supplementary Tables 1 and 2).

**Fig. 2:**
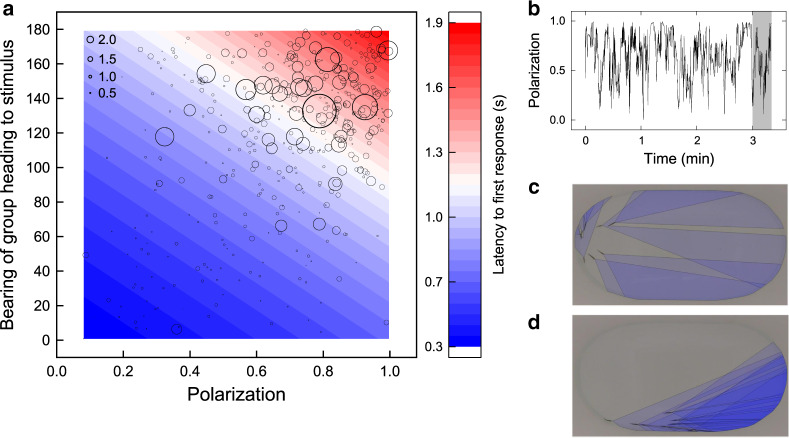
Variation in collective order affects the latency to first respond to the stimulus. **a** The effects of group polarization and bearing of the group heading to the stimulus on the latency of first responders to respond to the stimulus. Points represent the raw data with point sizes scaled relative to the latency to respond (seconds, scale bar displayed in the top left-hand corner). The colour gradient (blue to red) represents the predicted response latency (seconds) generated from a negative binomial GLMM with all other group-level parameters held at their mean value. **b** Example time series of a group’s polarization to illustrate temporal variation in collective order. The time series shows the 3 min before (unhighlighted) and 20 s following (highlighted in grey) a single stimulus presentation. **c** An illustration of the binocular visual range of a disordered group. **d** An illustration of the binocular visual range of a highly polarized group. In **c**, **d**, the blue regions highlight the area of the arena within the binocular visual range of the group, approximated with ray-casting methods (see Methods).

We next used a model comparison approach to investigate whether the groups’ position, movement and structure affected the speed that first responders responded to the stimulus. Consistent with the strong effect of the relative bearing to the stimulus on the likelihood of responding first, the bearing of the group heading to the stimulus was the strongest group-level predictor of response latency (RI = 1.00; Figs. 1b and 2a, and Supplementary Tables 3 and 4) with individuals in groups oriented towards the stimulus quicker to respond to the stimulus than those orientated away (Supplementary Fig. 4a). The first response to the stimulus became faster over repeated trials of the experiment (Fig. 1b, Cumulative days of testing). This effect is expected from training and acclimatisation to the stimulus^25^. Cumulative days of testing was also moderately correlated with an increase in group convex hull area and decrease in distance of the group centroid to the stimulus (Supplementary Table 5); however, neither of these parameters were important predictors of the latency to first respond to the stimulus (Fig. 1b). There was no statistically significant relationship between cumulative days of testing and group polarization (linear mixed model (LMM): cumulative days of testing: estimate (±SE) = 0.0062 ± 0.0038, Kenward–Roger F-test: *F*_1,281_ = 2.60, *P* = 0.11).

After controlling for the strong effects of the group’s orientation to the stimulus and cumulative days of testing, response latencies were also predicted by the groups’ collective order, measured as their polarization^1,2^ (Fig. 2a), which varied considerably in the times leading up to the stimulus presentations (Fig. 2b and Supplementary Fig. 5). The latency of the first fish to respond to the stimulus was faster if groups were more disordered (RI = 0.97, Figs. 1b and 2c, and Supplementary Fig. 4b) and slower if groups were more polarized with individuals orientated in the same direction as one another (Fig. 2d). This effect is an emergent property of individuals’ bearing towards the stimulus, which was more variable when the groups were disordered (Supplementary Fig. 6, inset, Spearman’s rank test: *r*_s_ = −0.88, *n* = 428, *P* < 2.2 × 10^−16^) and the minimum bearing angle to the stimulus among individuals was smaller when the group was less polarized (Supplementary Fig. 6, Spearman’s rank test: *r*_s_ = 0.76, *n* = 428, *P* < 2.2 × 10^−16^). As a result, the chance that the stimulus appeared within one of the individuals’ visual fields increased as group polarization decreased (Fig. 2c, d). This effect was strongest when considering the binocular visual regions of fish (Supplementary Movie 2) that are important for prey detection^26^, although the trend was still evident with a wider field of view that also includes the monocular region of the fish’s vision (Supplementary Fig. 7).

The potential effect of order on collective visual fields predicts that the latency of the first fish to respond should be fastest in polarized groups orientated towards the stimulus. In contrast to this prediction, there was no significant interaction term between the group’s polarization and their bearing towards the stimulus (GLMM (negative binomial): polarization × bearing of group heading to stimulus: estimate = 0.033 ± 0.033, likelihood ratio test (LRT): $\chi _1^2$ = 1.00, *P* = 0.32), but the two variables had independent main effects, so that the latency to respond was slower in more polarized groups across the range of bearings to the stimulus (Fig. 2a). However, when examining the latencies of all fish in the group to arrive at the stimulus, we did find a significant interaction between the group’s polarization and their bearing towards the stimulus (LMM: polarization × bearing of group heading to stimulus: estimate = 0.025 ± 0.0029, *F*_1,1963_ = 69.45, *P* < 2.2 × 10^−16^). The fastest individuals to arrive at the stimulus were those in polarized groups heading towards the stimulus, and the slowest were polarized groups heading away from the stimulus (Supplementary Fig. 8). The bearing towards the stimulus had less of an effect in disordered groups with low polarization, as predicted.

**Fig. 3:**
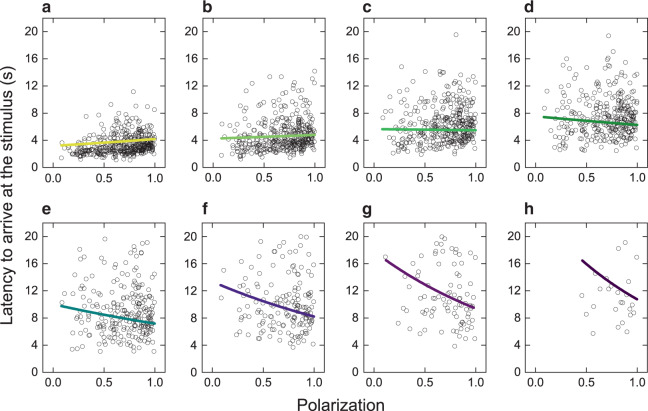
Variation in collective order affects the latency to arrive at the stimulus. The relationship between group polarization and the latency of an individual to arrive at the stimulus for different orders of arrival. Each panel shows data for fish with a different order of arrival at the stimulus: the **a** first (*n* = 428), **b** second (*n* = 406), **c** third (*n* = 376), **d** fourth (*n* = 315), **e** fifth (*n* = 241), **f** sixth (*n* = 165), **g** seventh (*n* = 87) and **h** eighth (*n* = 25) fish across presentations and trials (sample sizes are after the exclusion of *n* = 48 outliers from the model, see Methods). Points show the arrival latency of individual fish plotted against the median polarization of their group in the 0.5 s (13 frames) prior to the presentation. Lines show the predicted relationship between polarization and latency to arrive at the stimulus in each case, generated from a LMM (see Methods). The latency to arrive at the stimulus was calculated up to a maximum of 20 s (500 frames) following a presentation to capture responses to the stimulus only (see Methods). Hence, sample size for the sixth, seventh and eighth fish is small. However, a strong interaction effect between polarization and arrival order is apparent even when considering only the first to fifth fish to arrive.

The individual that responded to the stimulus first was much more likely to arrive first at the stimulus than expected by chance (one-sided binomial test: observed probability = 0.776, expected probability = 0.125, *n* = 428, *P* < 2.2 × 10^−16^) and the first fish to arrive at the stimulus consumed the food item in 68% of cases (Supplementary Fig. 9a). Thus, rapid responses to new private information resulted in preferential access to resources before competitors. To examine the consequences that collective order had on access to resources across all group members (not just the first individual to respond to the stimulus), we tested whether the latency of fish to arrive at the stimulus was predicted by the group’s polarization before the stimulus appeared and, further, whether this depended on the arrival order of individuals within their group to the stimulus after it was presented (ranked first to eighth fish). Although disorder appears to favour first responders, polarization may facilitate information transfer within groups^4,27^, improving response latencies for individuals that do not immediately respond to the stimulus. Confirming this, we found a highly significant interaction between group polarization and arrival order in the time taken to reach the stimulus (LMM: estimate = −0.026 ± 0.0032, Kenward–Roger F-test: *F*_1,1998_ = 67.9, *P* = 3.1 × 10^−16^, Supplementary Table 6); in other words, the effect of polarization was dependent on the arrival order. Although the time taken for the first and second fish to arrive at the stimulus was longer in more polarized groups, this effect reversed for the later arriving fish, which were faster when the group was more polarized (Fig. 3). This result is consistent with ordered group states facilitating the transmission of socially acquired information for fish that did not initially respond to the food through private information. As a result, the difference in arrival times between the first and last fish to arrive was larger when the groups were more disordered (low polarization), suggesting that group state can have a direct effect on whether resources are more evenly accessible to group members or are monopolized (Fig. 3).

In 32% of cases, the food item was consumed by an individual other than the first individual to arrive at the stimulus (Supplementary Fig. 9a), demonstrating that later arrivers could still access food. However, there was clear competition for food and potential for a large proportion of the food per trial being monopolized (Supplementary Fig. 9b). Individuals were repeatable in their order of arrival at the stimulus (LMM: LRT: Individual Identity Intercept: $\chi _1^2$ = 928.2, *P* < 2.2 × 10^−16^, Individual-level repeatability: *R* = 0.44, 95% confidence interval (CI_95%_) = 0.378–0.472, *P* < 2.2 × 10^−16^), including when the analysis was repeated without the first individuals to arrive (LMM: LRT: individual identity intercept: $\chi _1^2$ = 288.0, *P* < 2.2 × 10^−16^, individual-level repeatability: *R* = 0.29, CI_95%_ = 0.214-0.327, *P* < 2.2 × 10^−16^). This consistent variation between individuals in arrival order raises the potential for learning over repeated trials being stronger in those individuals more likely to arrive early, as they have a greater chance of being rewarded with food. This was indeed the case with a significant interaction between arrival order and cumulative days of testing in the time taken to arrive at the stimulus (LMM: estimate = 0.034 ± 0.0034, Kenward–Roger F-test: *F*_1,1992_ = 98.9, *P* < 2.2 × 10^−16^, Supplementary Table 7). The first to the fourth individuals to arrive had faster arrival latencies at the end of the experiment compared with the start, indicative of a learnt association of the stimulus with the food. Although the third and fourth individuals to arrive showed faster arrival latencies as the trials progressed, suggesting that they had enough access to the food item to form the association, they also benefited from social information by arriving at the stimulus sooner when the group was more polarized (Fig. 3 and Supplementary Table 6).

**Fig. 4:**
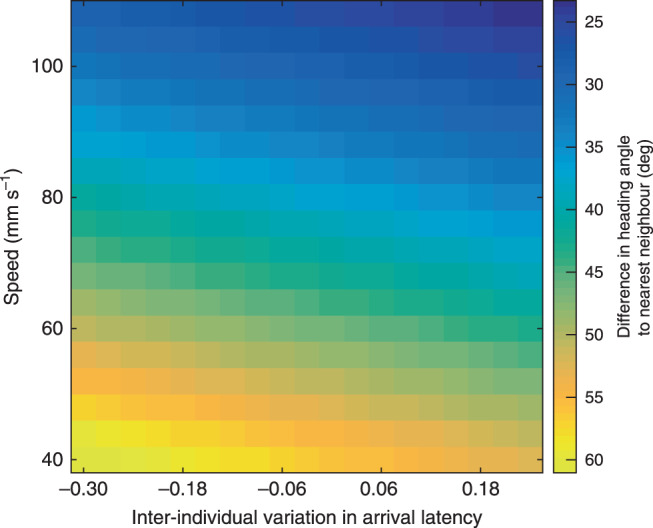
Individuals that are consistently faster to arrive at the stimulus maintain larger differences in the heading angle to their nearest neighbour outside of the foraging context. Heat map to show the relationship between the predicted difference in heading angle of individuals to their nearest neighbour as a function of inter-individual variation in arrival latency and swimming speed. Predictions are generated within the observed range for each parameter from a LMM with the mean heading difference to the nearest neighbour (averaged over every frame in the three minutes prior to the stimulus presentations) as the response variable, and the individual identity intercepts from a model of latency to arrive at the stimulus (Supplementary Table 6, excluding arrival order) and mean speed (averaged over every frame in the three minutes prior to the stimulus presentations) as main effects. The heat shows that within the observed range of mean swimming speeds, the heading difference of individuals to their nearest neighbour is larger for individuals that arrive consistently sooner at the stimulus (more negative values).

Theoretical models demonstrate how selection could give rise to the coexistence of individuals that differ consistently in their responsiveness to environmental and social information^28,29^. In addition to consistency between individuals in arrival order, there was consistent variation between individuals in their likelihood of responding first to the stimulus (GLMM (binomial), LRT: individual identity intercept: $\chi _1^2$ = 549.0, *P* < 2.2 × 10^−16^, individual-level repeatability, *R* = 0.38, CI_95%_ = 0.217–0.464, *P* < 2.2 × 10^−16^) and in their latency to arrive at the stimulus (LMM: LRT: individual identity intercept: $\chi _1^2$ = 157.3, *P* < 2.2 × 10^−16^, individual-level repeatability, *R* = 0.25, CI_95%_ = 0.188–0.314, *P* < 2.2 × 10^−16^) even after accounting for the effects of explanatory variables (Fig. 1a, b). These consistent differences between individuals may result in those that respond and arrive rapidly favouring a disordered, swarm-like group that maximizes their potential for acquiring the food item due to reduced competition with other group members. We thus examined the behaviour of individuals in the periods outside of the foraging context (i.e. in the intervals between presentations of the stimulus) and whether this was associated with the inter-individual differences during the stimulus presentations. After controlling for individuals’ average speed which is correlated to the average heading angle between neighbours^2^ (Spearman’s rank test: *r*_s_ = −0.34, *n* = 96, *P* = 0.006), individuals that typically arrived at the food source sooner maintained larger differences in the heading angle to their nearest neighbour when swimming in their groups outside of the foraging context (LMM: inter-individual variation in arrival latency: estimate = −0.15 ± 0.04, Kenward–Roger F-test: *F*_1,83_ = 13.44, *P* = 0.0004, Fig. 4). Therefore, individuals that benefited from being in less ordered groups during the stimulus presentations behaved in a manner that would contribute to disorder in their group’s collective motion.

We show that collective order can have opposing effects on the speed at which individuals react to resources in their environment, giving rise to conflict in the group structure that benefits different individuals. Individuals that are first to respond to (and arrive at) a new resource are faster to do so when the group is disordered and swarm-like, improving access to private information. In contrast, later responders arrive more quickly when the group is highly polarized, an effect that can be explained by more efficient transfer of social information in polarized groups^4,27^. This represents a fitness trade-off for individuals, depending on use of privately or socially acquired information. Inherent conflict between individuals for their preferred group order and competition for resources, therefore, provide a functional explanation for the variation we observe in a group’s collective behaviour.

## Methods

### Study animals

Three-spined sticklebacks (*G. aculeatus*, 27 ± 2.4 mm, mean ± SD, standard body length at the time of testing for *n* = 96 individuals), were collected from the river Cary, Somerset, UK (grid ref: ST 469 303) in September 2016 and were transported to the environmentally controlled fish laboratory at the University of Bristol, UK. The fish were housed in three glass tanks (70 cm (L) × 45 cm (W) × 37.5 cm (H)) of ~50 individuals for 10 months before testing and were fed daily with brine shrimp or defrosted frozen bloodworms (*Chironomid* sp. larvae). Photoperiod was on 11 : 13 h light : dark cycle and ambient temperature was maintained at 16 °C to prevent the fish from entering reproductive condition.

### Experimental set-up

Trials were conducted in an oval-shaped experimental arena (Supplementary Fig. 1; 133.5 (L) × 72 (W) with 62 cm high walls). The arena wall was constructed from white opaque foamed polyvinyl chloride (PVC, 3 mm thickness). The food stimulus could appear through one of four holes (3.5 mm ø) in the arena wall, which were located 5 cm high from the base of the arena. Fish were filmed from above with a Panasonic HC-VX980 video camera in 4 K (3840 × 2178 pixels) and a temporal resolution of 25 frames per second. The camera was fixed centrally above the arena with the lens 161 cm above, and perpendicular to, the water surface. Water was maintained at the same temperature as the holding tanks and was 10 cm deep. An opaque plastic curtain was hung from above camera height to below the height of the arena wall to minimize external disturbances and diffuse the overhead lighting to avoid reflections on the water surface. A second video camera (Panasonic HC-X920) was positioned above the arena and connected to two external monitors behind the curtain. The two monitors were positioned at opposite ends of the arena, allowing the experimenter to view the activity of the fish before presenting the food stimulus. Half of each monitor was covered with card to obscure the view of the half of the arena furthest from the experimenter. This ensured that the location and behaviour of the fish was blinded when the stimulus was presented (see below).

### Experimental protocol

One week prior to the experiment, 96 fish were randomly assigned to twelve groups of eight individuals. The assignment was carried out using a complete random block design: 12 fish were caught from 1 of the 3 holding tanks and each was randomly assigned to 1 of the 12 groups. This was repeated 8 times to form the 12 groups of 8. Each group included a minimum of two fish caught from each of the three tanks. This method of group assignment was designed to minimize variation between groups that could, e.g., be generated from bolder fish being caught earlier and all being assigned to the same group^30^. The fish were given 6 days to habituate in their groups in smaller glass holding tanks (70 (L) × 25 (W) × 37.5 (H) cm). Each holding tank was enriched with a horizontal piece of PVC tubing and an artificial plant.

Trials took place from Monday to Friday over 4 weeks between 31 July and 25 August 2017. Each week, the groups were randomly assigned to one of two sets of six groups. The two sets of groups were tested on alternate days. Hence, a group was never tested more than once every 2 days. Trials occurred between 09:45 and 16:00 each day and the order of testing among groups was randomized within each day.

At the start of a trial, all eight fish were netted into the centre of the arena and allowed 2 min to acclimatize. The experimenter then inserted the tip of a plastic pipette holding a single bloodworm into one of the four holes in the wall of the arena (randomly selected per presentation, Supplementary Fig. 1). The end of the pipette was wrapped in red PVC tape to provide a standardized (17 (L) × 3 (D) mm) visual stimulus that mimicked a bloodworm^31^. The visual stimulus was presented when all eight fish were on the opposite half of the arena to the selected hole, so that the experimenter was blind to the location and behaviour of the fish due to the obscured monitor display. The minimum possible distance between any individual and the stimulus when it was presented was 43 cm (Supplementary Fig. 1). When a fish swam within two body lengths of the stimulus as viewed on the monitor, the bloodworm was pipetted into the arena as a reward. The stimulus was removed once the bloodworm had been consumed or when all eight fish swam away from the stimulus towards the other side of the arena. If the reward was not immediately consumed, a new stimulus was not presented until the bloodworm had been eaten. During each trial, the stimulus was presented at the first opportunity after a minimum of a 3 min interval between successive presentations had elapsed and until six presentations had been performed (4.2 ± 0.9 min, mean ± SD, time gap between stimulus presentations within a trial). This time interval allowed the group to resume normal swimming behaviour between presentations, but varied depending on when the entire group was on the opposite side of the arena to the selected stimulus position. Limiting the number of presentations to six per trial meant that satiation effects were unlikely to influence the responses of the fish, as this species can consume over 50 bloodworms during a single feeding period^32^.

In 17 presentations, a fish appeared on the same side of the arena as the selected stimulus position during the presentation of the stimulus. In these cases, the stimulus presentation was repeated after 3 min. To maintain the minimum distance between the stimulus and the nearest fish, after reviewing the video footage, eight presentations were subsequently removed from our analyses, because the fish were quantitatively determined to be on the same side of the arena as the stimulus.

Following a trial, the group were returned to their holding tank. During the experimental period, all 12 groups were fed bloodworm in their holding tanks following the final trial of the day (Monday to Friday) and ad libitum during the weekend. This ensured that all fish had access to food and could maintain their health regardless of their performance in the trials. During the first week of trials, an individual in each of three groups was replaced with individuals naive to the experiment (one due to injury and two deaths of unknown cause) and given 24 h to habituate within their groups in the holding tanks prior to testing. All trials conducted prior to the replacements were excluded from the analyses. Thus, group membership was constant in the final dataset.

### Video processing and data extraction

Video files were converted from MP4 to M4V format in Handbrake (version 1.0.7, https://handbrake.fr/) and file resolution was reduced to 1920 × 1080 pixels to increase the speed of automated tracking. We used the automated two-dimensional tracking software idTracker^21^ to obtain the cartesian coordinate positions (*x*_i_, *y*_i_) for the centre-of-mass of each tracked individual (*i*) at each time step (*t*). To keep track of individual identities within the same group across the trials of the experiment, we re-used the fingerprint (individual specific) references generated by idTracker from the first trial of each group when processing video files from the subsequent trials. To reduce tracking noise, the trajectories of each fish were smoothed using a Savitzky–Golay filter with a span of 0.5 s (~13 frames) and a polynomial of 3 degrees in R package *Trajr*. Trajectory information for three stimulus presentations were not obtained due to corrupted video files. This resulted in tracked video footage for 468 presentations from 77 trials across the twelve groups.

### Statistical analyses

All statistical analyses were conducted in R (Version 3.4.3, R Development Core Team). The likelihood of being first to respond to the stimulus in a group, the response latency of the first responding individual, the arrival latency per individual, and the heading difference of individuals to their nearest neighbours were each analysed separately as response variables in mixed models (see Supplementary Methods for details on the identification of first responding individuals and the calculation of response variables). The statistical significance of fixed effects were tested using F-tests with Kenward–Roger approximated degrees of freedom in R package *pbkrtest* for LMMs and with LRTs in R package *lme4* for GLMMs. Estimates of inter-individual repeatability were generated in R package *rptR*.

To quantify the position and movement of individual fish and the group when the stimuli were presented, we calculated parameters from the trajectory data based on the position and change in position of the fish in the 0.5 s prior to presentation. Most positional and movement parameters were quantified by their median value over these 13 frames to reduce the weight of outliers and best capture the central tendency of the skewed distributions of the parameters.

To analyse the effects of the six positional and movement parameters on the likelihood that an individual is first in their group to respond to the stimulus, we used GLMMs with a binomial error distribution and logit link function. Response to the stimulus was considered a binary variable where 1 indicated the individual that was first in the group to respond and all other individuals in the group were given a score of zero. Fish identity was included as a random intercept to account for individual-level differences. As there could only be one first responder per group per presentation and the likelihood of being the first individual to respond was relative to other individuals in the group, we generated relative rather than aboslute values of each parameter. To generate relative measures, we divided the median value for each fish by the mean median value of all eight individuals in its group for each of the six parameters, except for relative proportion of time spent on the convex hull edge of the group, which we divided by the group mean proportion. We used these relative measures of movement and position as explanatory variables in our models. In addition, we included relative body length in our analysis due to reported correlations between body size and several factors that could influence responsiveness in fish (e.g., metabolic rate and feeding motivation^33^ and visual acuity^34^). We assessed the effect of each parameter by calculating model averaged coefficient estimates and 95% CIs from a set of candidate models that initially included all possible combinations of main effect terms. To ease interpretation of model averaged parameter estimates, all seven parameters were standardized (mean = 0, SD = 1) prior to analysis. We used corrected Akaike information criterion (AICc) values to evaluate our candidate models, owing to a low sample size-to-parameter ratio and used only those models with a cumulative weight of 95% to perform the final model averaging^35^ (Supplementary Table 2). As seven candidate models were <2 ∆AICc from the top-supported model and because we were interested in which factors have the strongest effect on likelihood of first response, we adopted a full model averaging approach^36^, implemented in R package *MuMIn*. In addition, we calculated the RI of the parameters by summing the Akaike weights for models in which they appear using the complete candidate set of models^35^. An RI tending towards 1 indicates that the parameter appears in the best supported models and an RI tending towards 0 indicates that the parameter appears in the least supported models^35^. To test whether individual identity accounted for important variation in the likelihood of first response to the stimulus, we compared the goodness-of-fit (deviance) between a binomial GLMM without any explanatory variables and fitted with a random intercept of fish identity to a general linear model with individual identity removed, using a LRT^37^. We also compared the goodness-of-fit between these models but including the four explanatory variables retained in the top-supported model based on AICc comparisons (Supplementary Table 2, model 1).

We took a similar approach to assess whether the five group-level positional and movement parameters explained variance in the latency of first responders to respond to the stimulus. Negative binomial GLMMs were used with the first responder’s identity nested within-group identity as a random effect. We performed the same AICc model averaging procedure outlined above from an initial set of candidate models including all possible combinations of the five group-level parameters and cumulative days of testing as main effects.

For fish that arrived at the stimulus within 20 s of presentation, we tested for an interaction between arrival order at the stimulus and group polarization on the latency to arrive at the stimulus using a LMM to examine the consequences that collective order had on access to resources for different group members. Group polarization was quantified in the 0.5 s prior to the stimulus presentation. Bearing of the group heading to the stimulus and cumulative days of testing (which had the strongest effects on the response latency of first responders), an interaction between bearing of the group heading to the stimulus and polarization and distance of the group centroid to the stimulus (which we expected to correlate positively with arrival latency) were also included in the model as main effects, and fish identity nested within-group identity was included as a random effect. Arrival latencies were log_10_ transformed prior to analysis. Post-transformation, the model residuals remained moderately right-skewed (skewness = 0.66 where values from −0.50 to 0.50 approximate symmetry) and further inspection revealed that this was owing to observations of arrival latencies close to the maximum of 20 s and underpredicted by the model. These observations also tended to be multivariate influential outliers in the model with greater than six times the mean Cook’s Distance^38^. To improve the fit to model assumptions, we re-ran the model of latency to arrive at the stimulus excluding observations with greater than six times the mean Cook’s Distance (*n* = 48) and we report the results from this analysis (*n* = 2043 observations) in the main text. However, qualitatively similar results were obtained when all observations (*n* = 2091) were included in the analysis (Supplementary Table 8). To test whether individual identity accounted for significant variation in arrival latencies, we compared the goodness-of-fit of the LMM to the same model with individual identity removed, using a LRT. We examined whether individuals were consistent in their order of arrival at the stimulus by LRT comparison of a LMM with arrival order as the response variable (sqrt transformed) and individual identity nested within-group identity as a random effect to the same model with individual identity removed. After confirming that individuals were consistent, we tested for an effect of learning that was dependent on arrival order by including an interaction between arrival order and cumulative days of testing in the model of latency to arrive at the stimulus.

Individual identity intercept values for the model of latency to arrive at the stimulus represent whether individuals arrived consistently sooner or later to the stimulus and hence should favour group disorder or group order. To test whether inter-individual variation in arrival latencies predicted the alignment behaviour of individuals to their nearest neighbours during periods outside of the stimulus presentations, we ran a LMM with the mean heading difference to the nearest neighbour (averaged over every frame in the three minutes prior to the stimulus presentations) as the response variable and the individual identity intercept values from a model of latency to arrive at the stimulus controlling for bearing of the group heading to the stimulus, distance of the group centroid to the stimulus, cumulative days of testing, polarization and an interaction between polarization and bearing of the group heading to the stimulus as a main effects (arrival order accounted for some of the inter-individual variation in the random intercepts and was excluded from the model). Mean speed (averaged over every frame in the three minutes prior to the stimulus presentations) was included as an additional main effect to control for the expected positive relationship between alignment and speed^2^ and group identity was included as a random effect.

All statistical models were inspected for under- and over-dispersion, and to ensure that they complied with assumptions of orthogonality, homoskedasticity, and normality of residuals. Model averaging approaches are generally robust to the effects of collinearity, nevertheless, we checked for issues related to collinearity among predictors by calculating Spearman’s rank correlations (*r*_s_, Supplementary Tables 5 and 9) and variance inflation factors (VIFs) in all our models. Only polarization and centroid speed showed evidence of collinearity (Supplementary Table 5); however, there was no evidence for strong (VIF > 3) multicollinearity in our models. For GLMMs, we used the R package *DHARMa* to interpret the model residuals. Statistical analyses were conducted on distance, time and related measurements in their raw units (i.e., frames and pixels—note these units remained consistent across all trials). For the reporting of data and model predictions in figures and tables the units of distance and time were converted to millimetres (where 1 mm was equal to 2.7 pixels) and seconds (where 1 s was equal to 25 frames), respectively.

### Ethics statement

All procedures regarding the use of animals in research followed United Kingdom guidelines and were approved by the University of Bristol Ethical Review Group (UIN UB/17/060).

### Reporting summary

Further information on research design is available in the Nature Research Reporting Summary linked to this article.

## Supplementary information

Supplementary Information

Reporting Summary

Description of Additional Supplementary Files

Supplementary Data 1

Supplementary Data 2

Supplementary Data 3

Supplementary Data 4

Supplementary Data 5

Supplementary Movie 1

Supplementary Movie 2

## Data Availability

The data that support these findings are available as Supplementary Data Files 1–5.

## Code availability

The code used to conduct the analyses are available on request from the authors.
