# Supplementary material for: Information can explain the dynamics of group order in animal collective behaviour
Source: Nat Commun. 2020 Jun 1;11:2737. doi: 10.1038/s41467-020-16578-x (PMC7264142; doi:10.1038/s41467-020-16578-x)
Supplement: Supplementary file 1 — Supplementary Information [file 41467_2020_16578_MOESM1_ESM.pdf]

Supplementary Information for

Information can explain the dynamics of group order in animal collective behaviour

Hannah E. A. MacGregor, James E. Herbert-Read & Christos C. Ioannou.

This file includes:

Supplementary Methods
Supplementary Figures 1 to 9
Supplementary Tables 1 to 10
Supplementary References

Supplementary Methods

Identification of first responders and calculation of response variables

We initially manually inspected the videos of each presentation and identified the fish that appeared to respond to the stimulus first (i.e. the first responder). This individual was identified as the fish that first showed a noticeable change in speed and direction towards the location of the stimulus. However, we then used the trajectory data to more quantitatively determine which individual responded first to the stimulus. To do this, we first calculated the change in position of fish *i* relative to the stimulus *s* between successive frames, such that

$$v_{i \to s}(t) = \frac{\sqrt{(x_s - x_i(t_{-1}))^2 + (y_s - y_i(t_{-1}))^2} - \sqrt{(x_s - x_i(t))^2 + (y_s - y_i(t))^2}}{t - t_{-1}} \quad (1)$$

where the position of fish *i* at time *t* is

$$\begin{pmatrix} x_i(t) \\ y_i(t) \end{pmatrix}$$

and the position of stimulus s is

$$\begin{pmatrix} x_s \\ y_s \end{pmatrix}$$

$v_{i \to s}$ effectively captures the velocity of a fish towards the stimulus, where negative values indicate the fish was moving away from the stimulus, and positive values indicate the fish was moving towards the stimulus (Supplementary Fig. 2). Within the 10 s after each stimulus presentation, we identified a threshold value of $v_{i \to s}$ that maximised the correspondence between the identity of the first responder based on manual inspection of the video recordings, and the identity of the first fish to exceed the threshold $v_{i \to s}$ across all presentations and trials. All presentations except one (which was removed) elicited a response by one or more individuals. Hence, for further analyses, we defined the first responder in the group as the first fish to reach the threshold $v_{i \to s}$= 157 mm s^{-1} (Supplementary Fig. 2), which resulted in an 85% match (399/467 presentations) to our manual inspection of the videos. In 21 cases, more than one individual reached the threshold in the same frame. For these individuals, we identified the local minimum of $v_{i \to s}$ that preceeded the time where $v_{i \to s}$ reached 157 mm s^{-1}. We then classified the first responder as the individual whose local minimum in $v_{i \to s}$ came before the others, as this represents the first discernable response of the fish to the stimulus (Supplementary Fig. 2). In six cases that remained where the local minimum in $v_{i \to s}$ occurred in the same frame for multiple individuals, the first responder was selected at random from these individuals.

The time point of the local minumum in $v_{i \to s}$ was also used for all first responders to quantify the response latency between the presentation of the stimulus and the first response to the stimulus. To maintain a consistent dataset across the analyses, we excluded 32 presentations where no local minimum in $v_{i \to s}$occurred prior to the fish reaching the threshold and seven presentations with multiple cases of individuals crossing trajectories before and after the stimulus presentation which resulted in inconsistencies in the tracking. Hence, for statistical

analyses, our final dataset consisted of trajectory information from 428 stimulus presentations from 77 trials.

To assess the potential effects on the time taken to acquire the potential food resource, we calculated the latency to arrive at the stimulus as the time difference between the presentation of the stimulus and the arrival of the individual at the stimulus (with arrival defined as the fish having a centre-of-mass position within two mean body lengths (54 mm) of the stimulus tip). This was calculated for all individuals regardless of whether they were first to respond or not. However, we restricted this analysis to only those fish that reached the stimulus within 20 s of presentation to avoid including fish swimming to the location where the stimulus was presented after completing a full lap of the arena. Hence, arrival latency was measured for 61% (2091 of 3424 potential cases) of individuals across presentations and trials. Where multiple fish arrived at the stimulus in the same frame, the order of arrival was selected at random among these individuals.

Positional and movement parameters

To assess how individual-level attributes influence the likelihood of an individual being the first individual to respond, we calculated six parameters:

(i) speed ($s_i(t)$): the distance travelled by fish *i* between two consecutive time frames,

$$s_i(t) = \frac{\sqrt{\left(x_i(t) - x_i(t_{-1})\right)^2 + \left(y_i(t) - y_i(t_{-1})\right)^2}}{t - t_{-1}} \quad (2)$$

(ii) distance to the stimulus ($d_{i \to s}(t)$): the distance between the position of fish *i* and the stimulus *s*,

$$d_{i \to s}(t) = \sqrt{(x_i(t) - x_s(t))^2 + (y_i(t) - y_s(t))^2} \quad (3)$$

(iii) bearing to the stimulus ($\theta_{i \to s}(t)$): the angle between the vector heading of fish *i* and the direction from fish *i* to the stimulus,

$$\cos\theta_{i \to s}(t) = \frac{\vec{v}_i(t).\vec{v}_{i \to s}(t)}{|\vec{v}_i(t)||\vec{v}_{i \to s}(t)|} \quad (4)$$

where the vector heading of fish *i* is defined by the vector difference in the centre of mass of fish *i* between consecutive frames

$$\vec{v}_i(t) = \begin{pmatrix} x_i - x_{i-1} \\ y_i - y_{i-1} \end{pmatrix} \quad (5)$$

(iv) proportion of time on the convex hull edge of the group: i.e. the proportion of frames that fish *i* spends on the convex hull edge of the group. We used the *chull* function in R package *splancs* to calculate the minimum length two-dimensional convex hull, whose vertices are the positions of fish, and which contains all eight fish in a group. The proportion of time on the convex hull edge of the group[1] was calculated as the sum of the number of frames where fish *i* was a vertex of the convex hull divided by 13 (the number of frames in 0.5 s).

(v) distance to the group centroid ($d_{i \to c}(t)$): the distance between the position of fish i and the mean position of all eight individuals in the group (group centroid), where the position of the group centroid c for N individuals is defined as

$$\begin{pmatrix} x_c\ (t) \\ y_c\ (t) \end{pmatrix} = \frac{1}{N} \begin{pmatrix} \sum_{i=1}^{N} x_i \\ \sum_{i=1}^{N} y_i \end{pmatrix} (t) \tag{6}$$

and

$$d_{i \to c}(t) = \sqrt{(x_i(t) - x_c(t))^2 + (y_i(t) - y_c(t))^2} \tag{7}$$

(vi) visual occlusion: the proportion of a fish's visual field occupied by other individuals. This measures assumes that the fish swim on the same horizontal plane, and each fish has 360 degrees of visual field around its centre-of-mass. We first estimated the body length of all individuals using ImageJ version 1.52 (https://imagej.nih.gov/ij/download.html) from full resolution still images captured from the first trial of each group. Measurements were calibrated with a scale bar placed within the arena prior to the experiment and subsequently removed. Two measurements were recorded for each fish from separate frames, and the mean body length (rounded to the nearest mm) was retained. Based on these body length estimates and the vector heading of all eight fish in the group, we calculated the number of 1 degree segments of a fish's visual field that were occupied by at least one neighbour implemented in MATLAB R2018b. We then divided this by 360 to determine the proportion of their visual field occuiped by conspecifics[2].

An individual's position relative to the front of the group[3] was not included as a measure as it is only a meaningful parameter when the group is highly polarized, and the groups often had low levels of polarization when the stimulus was presented (Supplementary Fig. 5).

To examine the relationships between group behaviour and the latency of the first individual to respond to the stimulus, and separately the latency to arrive at the stimulus for each individual, we calculated five group-level parameters:

(i) convex hull area: the area enclosed by individuals on the convex hull edge of the group (see prop. time on convex hull edge),
(ii) bearing of the group heading to the stimulus ($\theta_{c,c \to s}$): the angle between the vector heading of the group centroid and the direction between the group centroid and the stimulus,

$$\cos \theta_{c,c \to s} = \frac{\vec{v}_c(t).\, \vec{v}_{c \to s}(t)}{|\vec{v}_c(t)||\vec{v}_{c \to s}(t)|} \tag{8}$$

where velocity of the group centroid towards the stimulus is defined by

$$v_{c\to s}(t) = \frac{\sqrt{(x_s - x_c(t_{-1}))^2 + (y_s - y_c(t_{-1}))^2} - \sqrt{(x_s - x_c(t))^2 + (y_s - y_c(t))^2}}{t - t_{-1}} \tag{9}$$

(iii) distance of the group centroid to the stimulus: the distance between the group centroid and the stimulus

$$d_{c\to s} = \sqrt{(x_s(t) - x_c(t))^2 + (y_s(t) - y_c(t))^2} \tag{10}$$

(iv) centroid speed: the distance travelled by the group centroid between two consecutive frames

$$v_c = \frac{\sqrt{(x_c(t) - x_c(t_{-1}))^2 + (y_c(t) - y_c(t_{-1}))^2}}{(t - t_{-1})} \tag{11}$$

(v) polarization[4,5]: also known as the order parameter (Op), a measure of angular alignment from 0 (non-polarized) to 1 (perfectly aligned)

$$Op = \frac{1}{N}\left|\sum_{i=1}^{N} \vec{u}_i\right| \tag{12}$$

where $\vec{u}_i$ represents the unit vector of individual i and N represents the number of fish in the group.

Estimating visual information

To evaluate the relationship between group polarization and the potential availability of visual information to the group in the 0.5 s (13 frames) prior to the stimulus presentations, we calculated the proportional area of the arena within the visual range of at least one group member after accounting for visual occlusion due to group members' positions and orientations. To do this, we used a ray casting method[2] implemented in MATLAB R2018b based on three different field of view sizes: (i) a binocular field of view (associated with prey detection[6]), approximated as 30° based on recently reported values for two freshwater shoaling species[7]; (ii) a 180° field of view, and (iii) a 330° field of view. For each field of view size, rays were cast within the field of view every 0.1 degrees from the frontal-dorsal region of each fish and either terminated at points on the arena boundary or upon intersection with a neighbouring fish (characterised by line segments determined through fish's positions, orientations and lengths). These termination points were used to construct the minimal enclosing polygon that represented the area of the arena within the visual range of at least one group member (see Fig. 2c, d). This area was then divided by the total area of the arena to calculate the proportional area of the arena within the visual range of the group.

Heading difference to nearest neighbour

To quantify individuals' tendency to align with their neighbours outside of the time when the stimuli were presented, we additionally calculated the heading difference of each

individual to their nearest neighbour for every frame in the three minutes prior to the stimulus presentations according to the formula

$$\cos\theta_{ij} = \frac{\vec{v}_i(t).\vec{v}_j(t)}{|\vec{v}_i(t)||\vec{v}_j(t)|} \quad (13)$$

where fish *j* is the individual with the minimum distance to the focal individual i, and $\vec{v}_i$ and $\vec{v}_j$ are the velocities of fish *i* and *j* respectively.

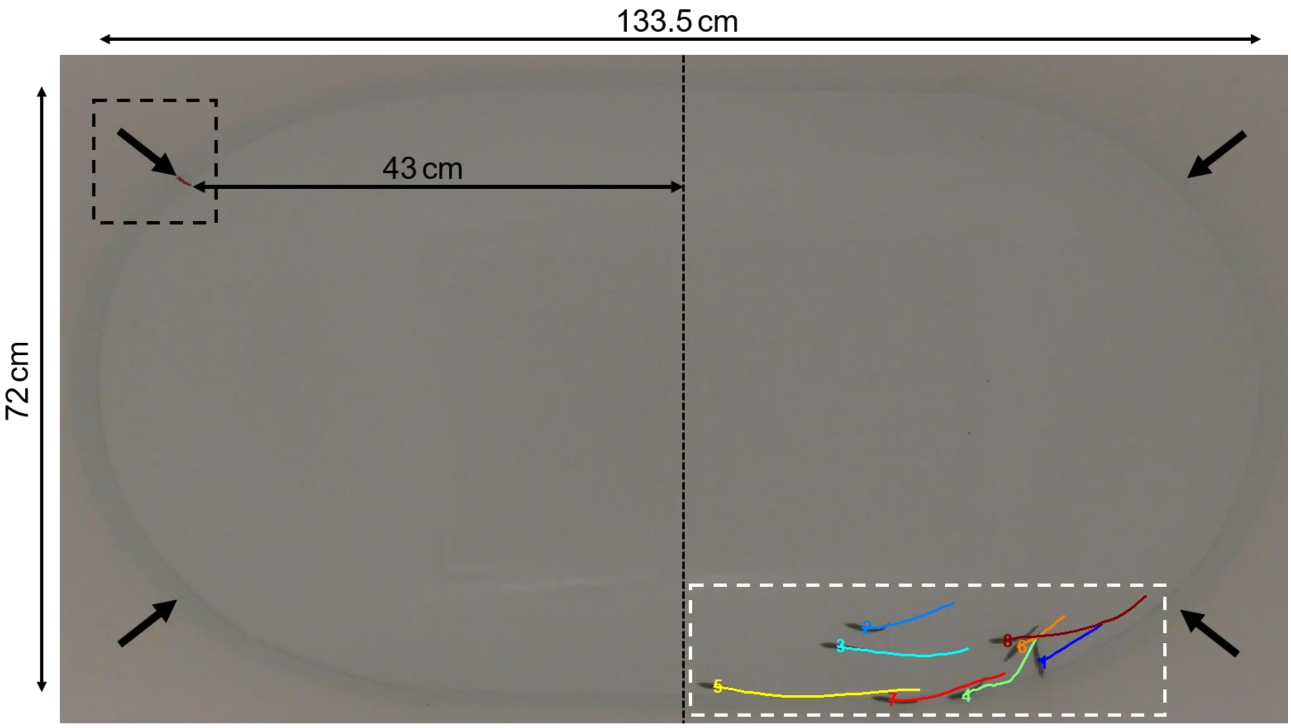

Supplementary Figure 1 | The experimental set-up from above showing a group of eight individuals with tracking highlighted (generated with idTracker[8]). The length and width of the oval-shaped experimental arena are reported outside of the image. The eight fish (within the white dashed box) are pictured on the opposite half of the arena to the location of the red visual stimulus (within the black dashed box). The black dotted line marks the division between the two halves of the arena and, hence, the region up to which the experimenter was blinded to the location and behavior of the fish due to the partially obscured monitor displays. The minimum possible distance between an individual and the stimulus was 43 cm. Black arrows indicate the four possible locations from where the stimulus could appear (randomly selected per presentation). The individual identities of all fish in the groups were retained across the multiple trials of the experiment when the trajectory data were generated.

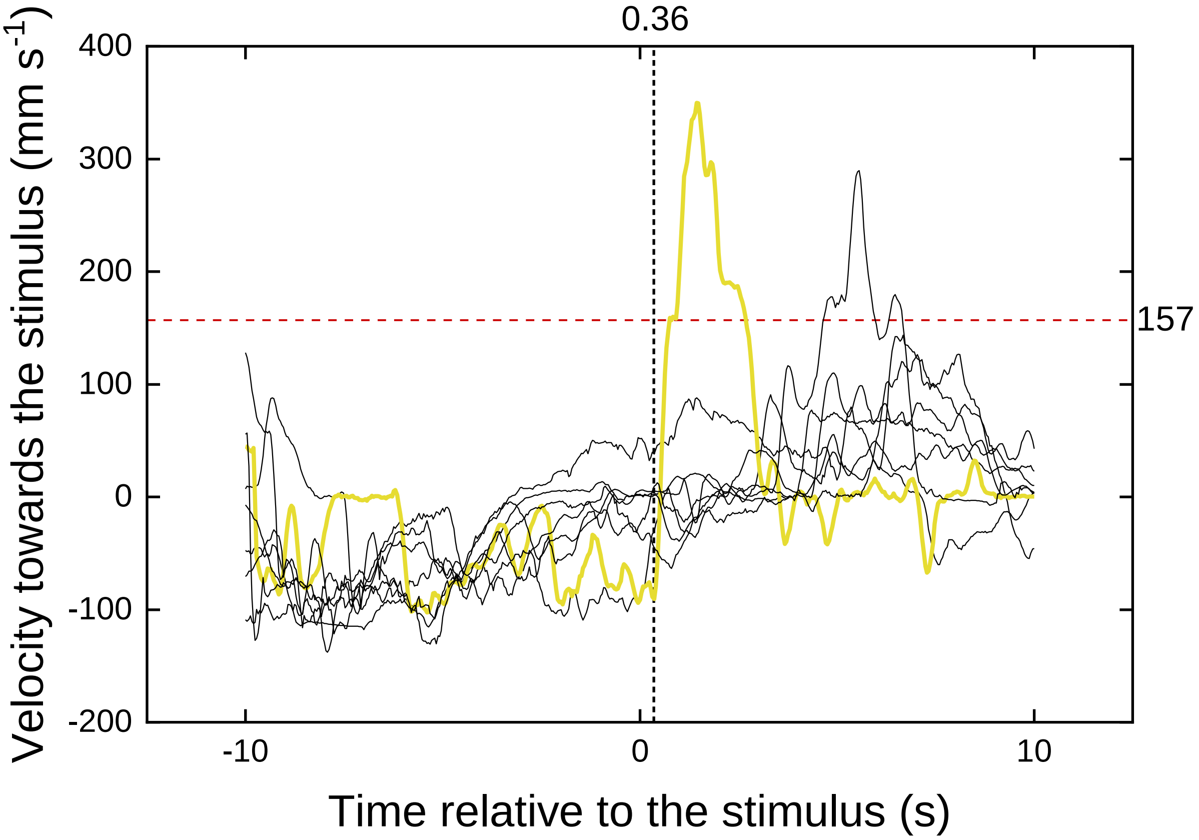

Supplementary Figure 2 | Example time series of the velocity towards the stimulus $v_{i\rightarrow s}(t)$ for 10 s prior and 10 s following a stimulus presentation (which occurs at time relative to the stimulus = 0). Each solid line shows an individual fish (yellow is the fish identified as the first responder to the stimulus, and black depicts the other seven group members). Positive and negative velocities indicate that fish were moving towards or away from the stimulus, respectively. The horizontal dashed red line is the threshold $v_{i\rightarrow s}$ (157 mm s^{-1}) used to identify first responders across presentations and trials (see Supplementary Methods). The vertical dashed black line (at 0.36 s) intersects the first local minimum in $v_{i\rightarrow s}$ in the time prior to when $v_{i\rightarrow s}$ reaches 157 mm s^{-1} , and this time was taken as the fish's response latency

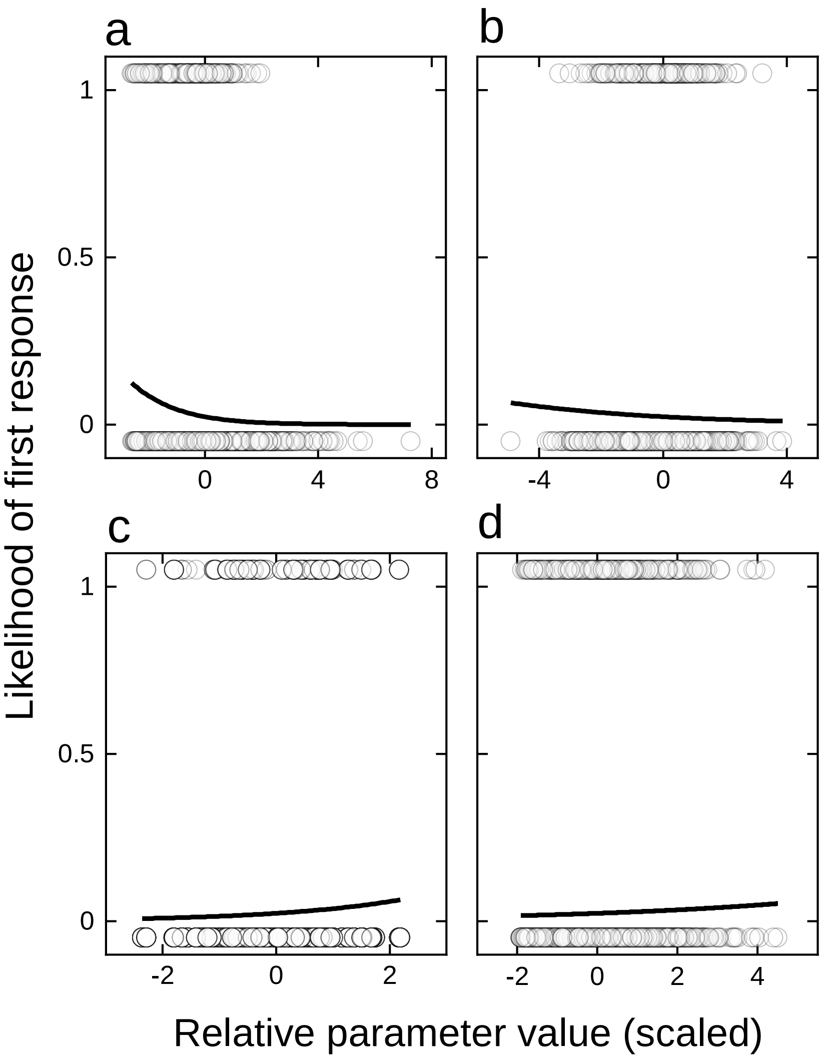

Supplementary Figure 3 | Predicted likelihood that an individual was the first to respond as a function of relative individual-level parameters: a, relative bearing to stimulus, b, relative distance to stimulus, c, relative body length, and d, relative speed. All parameters were calculated relative to the other individuals in the group (see Methods) and were scaled (mean = 0, sd =1) in the models. Lines show the predicted likelihoods within the observed range for each parameter and are generated from the top supported candidate binomial GLMM based on AICc model comparisons, which retained all four parameters shown (Supplementary Table 2, model 1). In each case, predicted values are generated with all other terms held constant at their mean value. Observed binomial response data (1: first responder, 0: all other fish) are plotted vertically offset from zero and one.

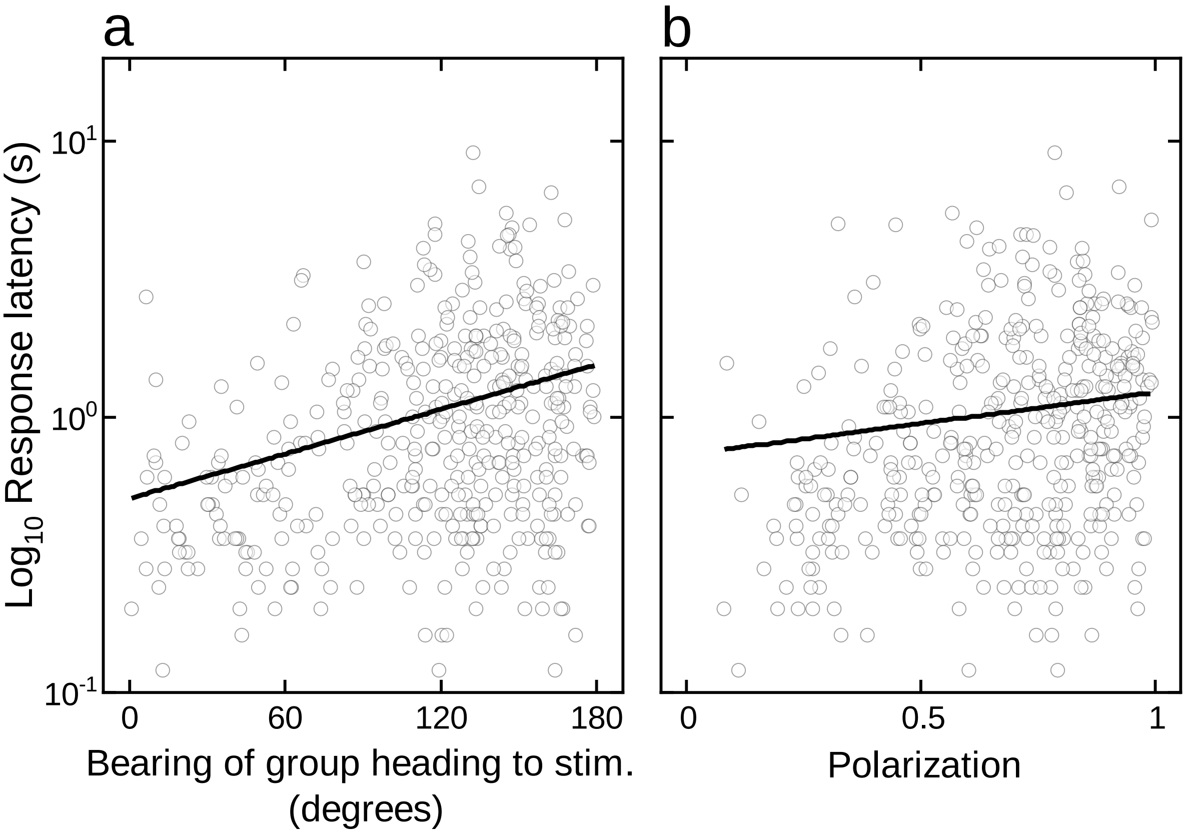

Supplementary Figure 4 | The positive effects of a, the bearing of the group heading to the stimulus (degrees) and b, group polarization on the response latency of first responders. Points depict the median values of the parameters in the 13 frames (0.5s) prior to the stimuli. The black trend lines show the predicted fitted values from negative binomial GLMMs including terms retained in the top supported candidate model of response latency of first responders based on AICc model comparisons (Supplementary Table 4, model 1). In each case, all other terms were held constant at their mean values for the generation of predictions.

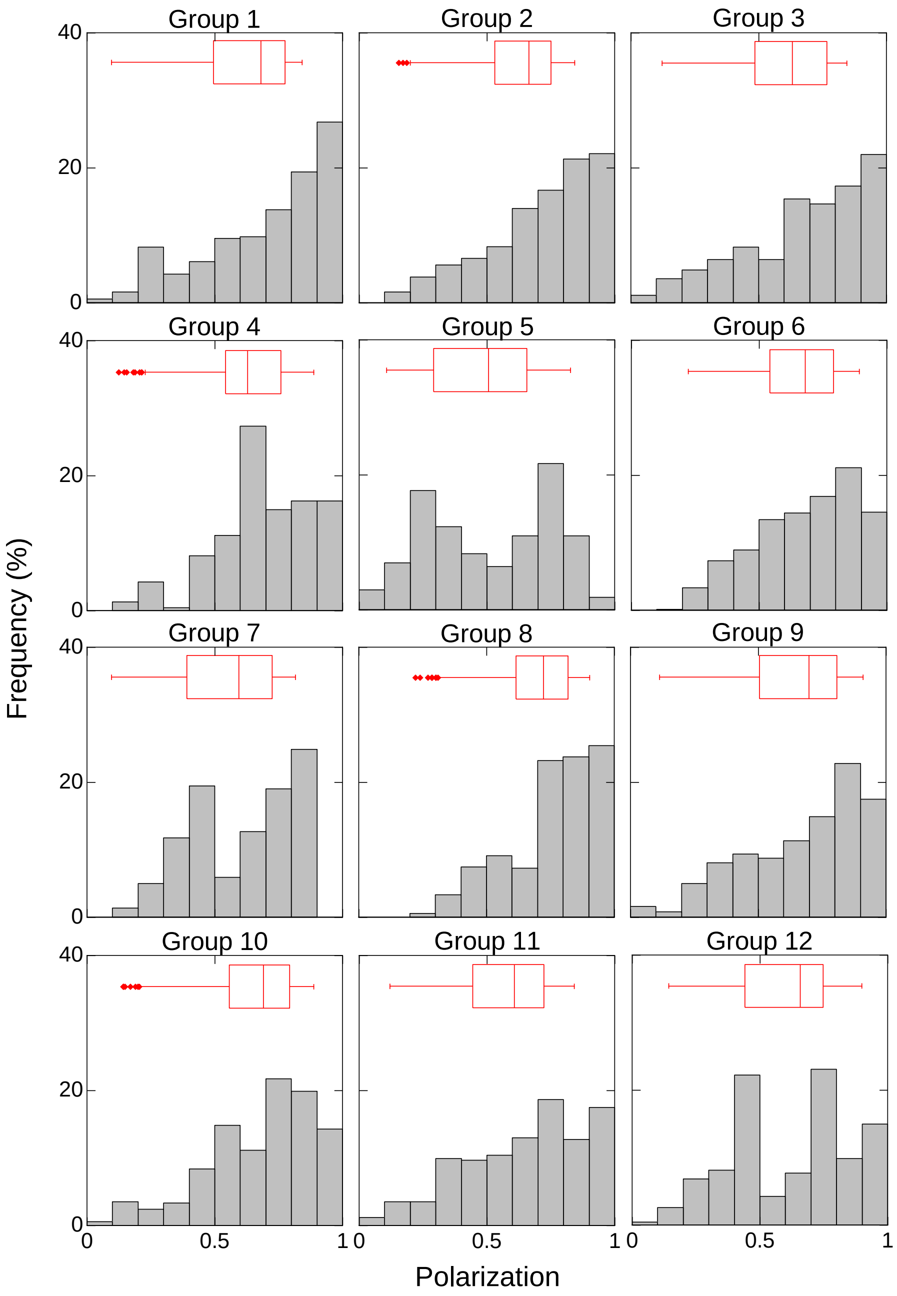

Supplementary Figure 5 | Percentage frequency distributions of group polarization prior to the presentations of the stimuli shown for each experimental group. The data are pooled over the 0.5 s (13 frames) prior to all the stimulus presentations for each group (Group 1 (n = 29), Group 2 (n = 48), Group 3 (n = 41), Group 4 (n = 18), Group 5 (n = 29), Group 6 (n = 63), Group 7 (n = 17), Group 8 (n = 42), Group 9 (n = 48), Group 10 (n = 42), Group 11 (n = 33), Group 12 (n = 18)). Above each distribution, the red box plot defines the interquartile range (enclosed by the box) and the median value (vertical red line) of the data for each group. The box plot whiskers delineate data within 1.5× the interquartile range outside the box. Red diamonds show data points beyond the range of the whiskers.

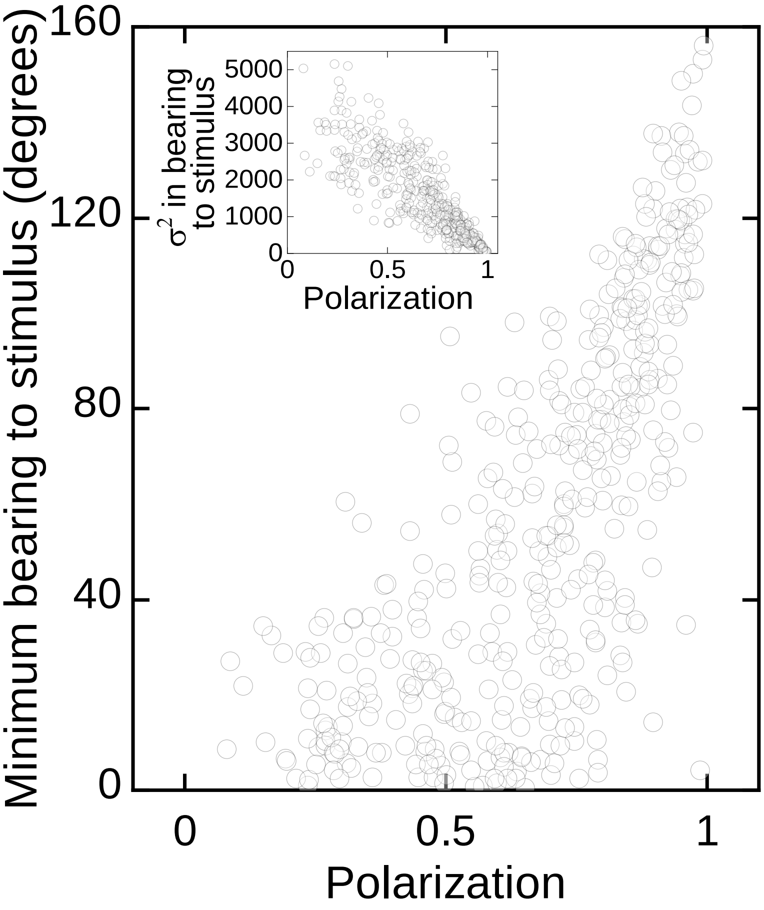

Supplementary Figure 6 | Relationship between group polarization and the within-group minimum bearing to the stimulus. Points depict the median group polarization in the 0.5 s (13 frames) prior to the stimuli plotted against the smallest bearing to the stimulus when the group polarization was at the median group polarization value (n = 428). As polarization increases, the minimum bearing to the stimulus in the group becomes larger, on average, because the variance in the bearing to the stimulus among group members decreases (shown in the inset).

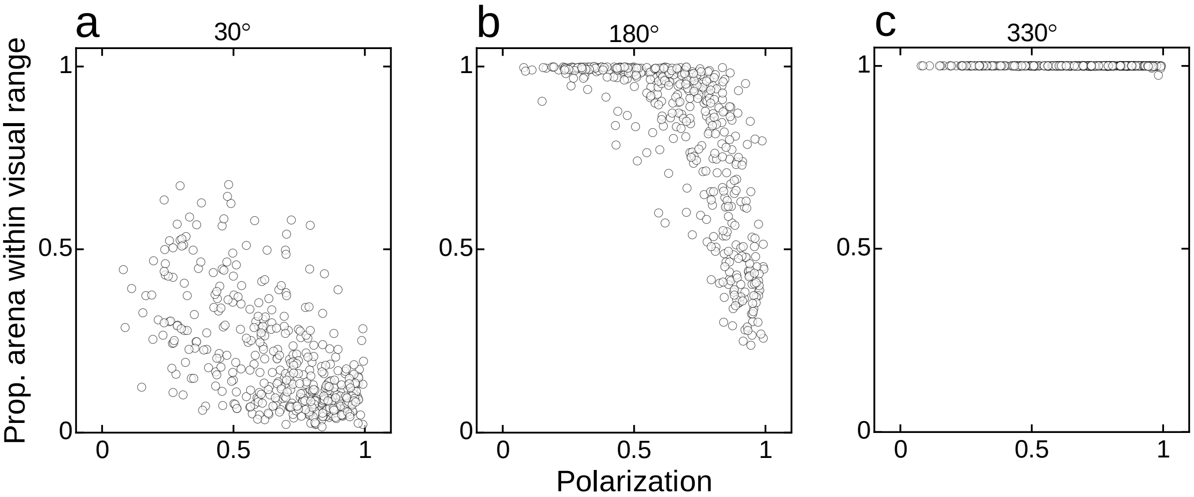

Supplementary Figure 7 | The associations between group polarization and the proportion of the arena within the visual ranges of the groups for different sized fields of view. a, A 30° field of view (i.e. a binocular field of view that is associated with acute vision[7]), **b**, an 180° angle field of view (i.e. that incorporates binocular and some monocular vision), and **c**, a 330° angle field of view (that excludes a 30° blind angle behind each fish). In each case, the proportion of the arena within the visual range of the group is approximated with ray casting methods accounting for visual occlusion between neighbours. All values are median values taken from the 0.5 s prior to the presentation of the stimuli.

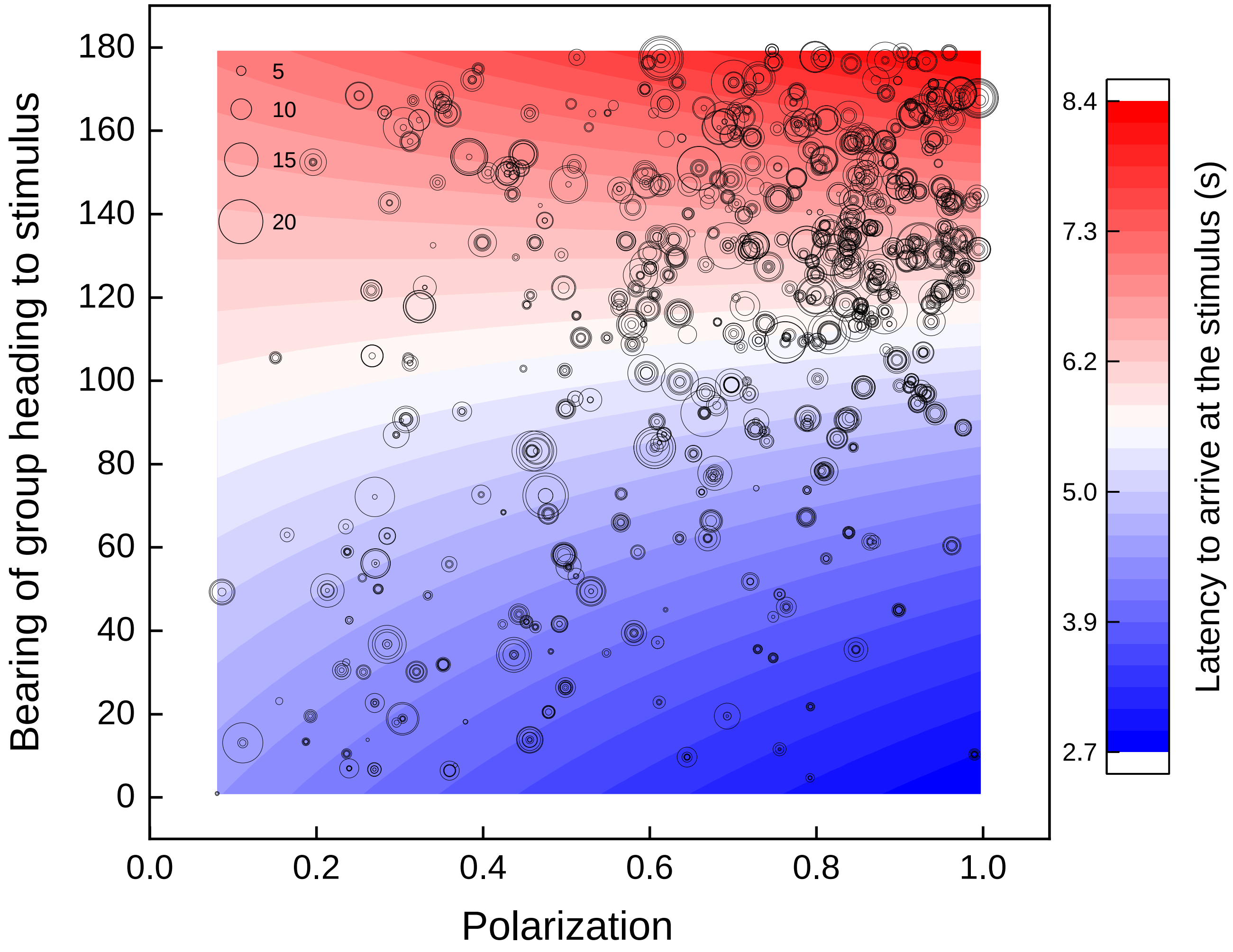

Supplementary Figure 8 | The effect of an interaction between group polarization and bearing of the group heading to the stimulus on the latency to arrive at the stimulus. The bubbles represent the raw data for individual fish with bubble sizes scaled relative to their latency to arrive (seconds, scale bar displayed in the top left-hand corner). The colour gradient (blue to red) represents the predicted latency to arrive at the stimulus (seconds) generated from a LMM controlling for the distance of the group centroid to the stimulus, cumulative days of testing, arrival order, and interaction between polarization and arrival order.

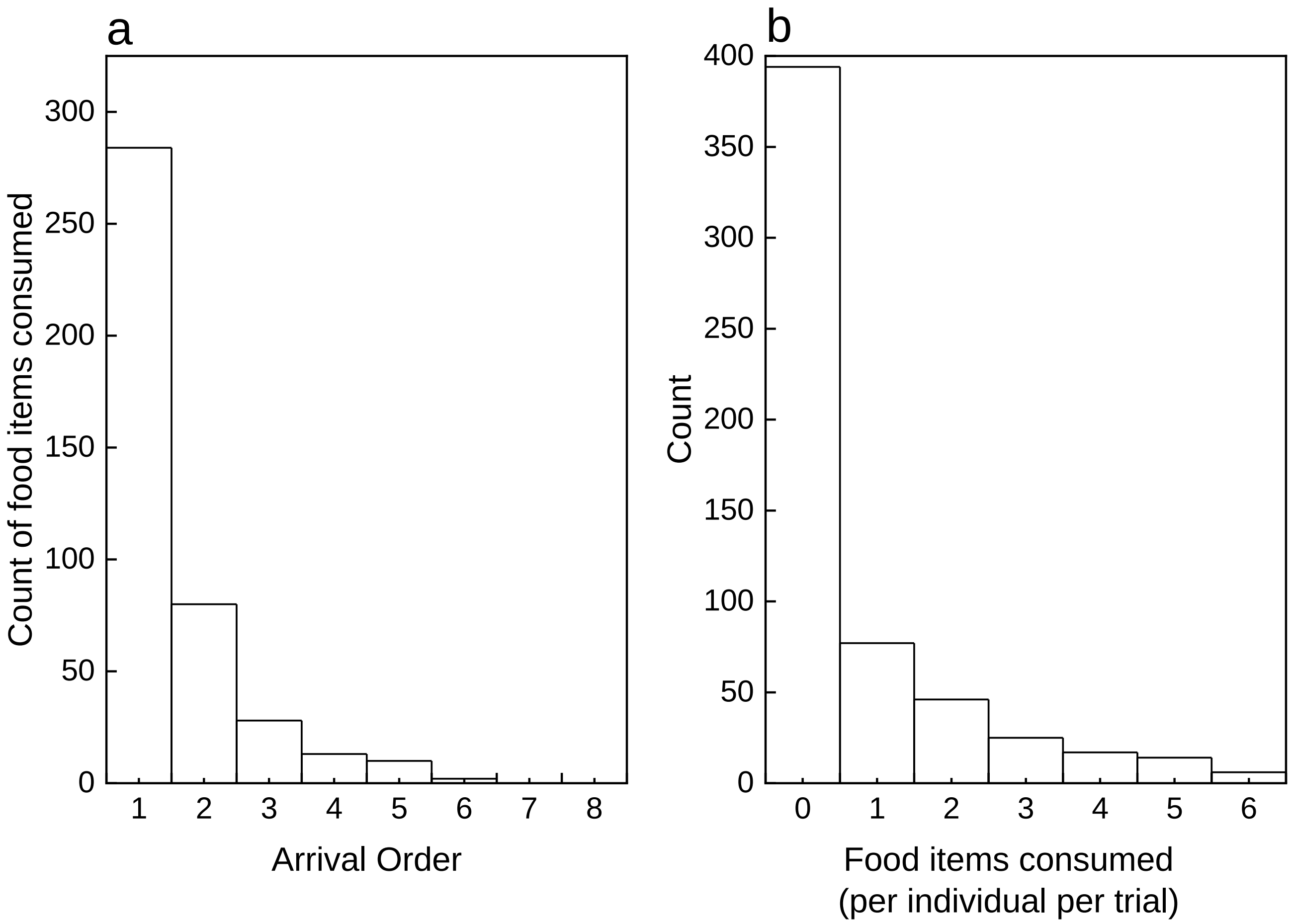

Supplementary Figure 9 | Distributions to show the consumption of food items at the stimulus. In **a**, the histogram shows the distribution of food items consumed by the order that an individual arrived at the stimulus, and in **b**, the histogram shows the distribution of the total number of food items consumed per individual per trial. Stimulus presentations were limited to 6 presentations per trial with a single bloodworm delivered per presentation to avoid satiation effects. Hence the maximum number of food items consumed per individual is six. The data presented are based on n = 418 presentations where the feeding fish could be identified from the tracked video footage.

Supplementary Table 1 | Standardized model averaged coefficient estimates from candidate binomial GLMMs to determine the predictors of the likelihood an individual is the first to respond. All parameters are calculated relative to the other individuals in the group (by dividing individuals' median values from the 0.5 s prior to the presentation of the stimulus by the mean median values of all eight group members, or, in the case of relative body length and relative proportion of time on the convex hull edge of the group, by dividing by the mean of all eight group members). Standardized model averaged coefficient estimates (based on the 95% candidate set of models, Supplementary Table 2) are reported with the upper and lower 95% confidence limits in brackets for each parameter. The relative importance (RI) of each parameter is the sum of the Akaike weights over the models in which that parameter appears (based on the complete candidate set of models including all possible combinations of main effect terms).

Parameters	Estimate ($CI_{95\%}$)	RI
Relative body length	0.28 (-0.347, 0.910)	0.59
Relative proportion of time on convex hull edge	0.04 (-0.092, 0.167)	0.42
Relative bearing to stimulus	-0.69 (-0.833, -0.541)	1.00
Relative distance to stimulus	-0.21 (-0.345, -0.077)	0.98
Relative speed	0.18 (0.050, 0.311)	0.96
Relative visual occlusion	0.00 (-0.086, 0.079)	0.28
Relative distance to centroid	0.01 (-0.086, 0.112)	0.31

Supplementary Table 2 | The 95% candidate set of models used in model-averaging to generate estimates of the effect sizes of body length and six spatial and movement parameters on the likelihood an individual is the first to respond. Models are ranked in order of AICc (smallest to largest, Model 1 R^2 conditional (delta method) = 0.43). Standardized model parameter estimates are reported for the parameters that occur in each model. The number of parameters (k), the log-likelihood, and the Akaike weight are also reported. The AICc value for the 'null' model is presented for comparison (bottom row). Individual identity was included as a random effect in all candidate models. Individuals consistently differed in their likelihood to respond first to the stimulus, and this repeatability was stronger in the null model without any explanatory variables (GLMM (Binomial), LRT: Individual Identity Intercept: $\chi^2 = 683.0$, $P < 2.2 \times 10^{-16}$).

	Standardized Model Parameter Estimates											
Model	Relative body length	Relative proportion of time on convex hull edge	Relative bearing to stimulus	Relative distance to centroid	Relative distance to stimulus	Relative visual occlusion	Relative speed	*k*	logLik	AICc	ΔAICc	weight
1	0.475		-0.687		-0.216		0.185	6	-890.1	1792.3	0.00	0.15
2	0.474	0.088	-0.686		-0.213		0.182	7	-889.3	1792.6	0.31	0.13
3			-0.688		-0.216		0.184	5	-891.5	1793	0.74	0.11
4		0.088	-0.686		-0.213		0.181	6	-890.6	1793.3	1.03	0.09
5	0.474		-0.688	0.061	-0.200		0.184	7	-889.7	1793.5	1.23	0.08
6	0.472		-0.688		-0.211	-0.039	0.183	7	-889.9	1793.9	1.67	0.07
7			-0.688	0.062	-0.199		0.182	6	-891.1	1794.2	1.94	0.06
8	0.475	0.094	-0.686		-0.215	0.012	0.183	8	-889.3	1794.6	2.30	0.05
9	0.474	0.083	-0.686	0.007	-0.212		0.182	8	-889.3	1794.6	2.32	0.05
10			-0.688		-0.210	-0.041	0.182	6	-891.3	1794.6	2.37	0.047
11		0.093	-0.686		-0.214	0.009	0.181	7	-890.6	1795.3	3.02	0.034
12		0.083	-0.687	0.008	-0.211		0.181	7	-890.6	1795.3	3.03	0.034
13	0.473		-0.688	0.057	-0.200	-0.007	0.183	8	-889.7	1795.5	3.23	0.031
14			-0.689	0.057	-0.200	-0.009	0.182	7	-891.1	1796.2	3.94	0.021
15	0.475	0.088	-0.686	0.014	-0.212	0.016	0.183	9	-889.2	1796.6	4.29	0.018
16		0.087	-0.686	0.014	-0.211	0.014	0.181	8	-890.6	1797.3	5.01	0.013
17	0.469		-0.701		-0.223			5	-894.3	1798.7	6.44	0.006
18	0.467	0.094	-0.699		-0.220			6	-893.4	1798.8	6.49	0.006
Null								2	-948.5	1901.1	108.8	

Supplementary Table 3 | Standardized model averaged coefficient estimates from candidate negative binomial GLMMs to examine the predictors for the response latency of first responders. All parameters except for cumulative days of testing are the median values taken from the 13 frames (0.5 s) prior to each stimulus presentation. Standardized model averaged coefficient estimates (based on the 95% candidate set of models, Supplementary Table 4) are reported for each parameter with the upper and lower 95% confidence limits in brackets. The relative importance (RI) of each parameter is the sum of the Akaike weights over the models in which that variable appears (based on the complete candidate set of models including all possible combinations of main effect terms). Despite a correlation between group centroid speed and polarization (Supplementary Table 5), qualitatively similar results were produced after the removal of group centroid speed from the candidate sets of models (Supplementary Table 10).

Parameter	Estimate ($CI_{95\%}$)	RI
Bearing of group heading to stimulus	0.28 (0.198, 0.354)	1.00
Convex hull area	-0.01 (-0.065, 0.043)	0.36
Distance of group centroid to stimulus	0.03 (-0.047, 0.107)	0.54
Polarization	0.14 (0.031, 0.252)	0.97
Centroid speed	-0.03 (-0.137, 0.068)	0.50
Cumulative days of testing	-0.29 (-0.373, -0.208)	1.00

Supplementary Table 4 | The 95% candidate set of models used in model-averaging to generate estimates of the effect sizes of five group-level positional and movement parameters and cumulative days of testing on the response latency of first responders. Models are ranked in order of AICc (smallest to largest, Model 1 R^2 conditional (delta method) = 0.37). Standardized model parameter estimates are reported for the parameters that occur in each model. The AICc value for the null model is presented for comparison (bottom row). Models are ranked in order of AICc (smallest to largest). Model term estimates are reported for parameters that occur in each model. The number of parameters (*k*), the log-likelihood, and the Akaike weight are also reported. The AICc value for the 'null' model is presented for comparison (bottom row). First responder identity nested within group identity was included as a random effect in all candidate models.

	Standardized Model Parameter Estimates										
Model	Cumulative days of testing	Bearing of group heading to stimulus	Convex hull area	Distance of group centroid to stimulus	Polarization	Centroid speed	*k*	logLik	AICc	ΔAICc	weight
1	-0.28	0.28		0.06	0.12		8	-1776.39	3569.12	0.00	0.23
2	-0.28	0.26		0.05	0.17	-0.07	9	-1775.54	3569.51	0.39	0.19
3	-0.31	0.27			0.19	-0.09	8	-1776.73	3569.80	0.69	0.16
4	-0.31	0.29			0.12		7	-1778.04	3570.35	1.23	0.12
5	-0.29	0.27	-0.04		0.17	-0.08	9	-1776.09	3570.61	1.49	0.11
6	-0.29	0.29	-0.05		0.11		8	-1777.16	3570.66	1.55	0.10
7	0.28	-0.02	0.05	0.11		0.28	9	-1776.21	3570.86	1.74	0.09
Null							4	-1852.76	3713.62	144.50	

Supplementary Table 5 | Spearman's rank correlations among five group-level parameters and cumulative days of testing, tested for their effects on the response latency of first responders. All data except for cumulative days of testing were median values taken from the 0.5 s prior to each stimulus presentation. Group centroid speed and polarization showed moderate collinearity.

	Distance of group centroid to stimulus	Centroid speed	Polarization	Bearing of group heading to stimulus	Cumulative days of testing
Convex hull area	-0.52	-0.09	-0.26	-0.02	0.29
Distance of group centroid to stimulus		0.03	0.11	-0.05	-0.35
Centroid speed			0.72	0.07	0.00
Polarization				0.32	-0.03
Bearing of group heading to stimulus					0.13

Supplementary Table 6 | Model predictions for the effect of the interaction between group polarization (O*p*) and order of arrival at the stimulus on the latency to arrive at the stimulus. Predictions are generated from a model of latency to arrive at the stimulus (R^2 conditional = 0.65) controlling for the significant effects of the bearing of the group heading to the stimulus (LMM: Estimate = 0.097 ± 0.0037, Kenward-Roger F-test: $F_{1,1965}$ = 695.7, $P < 2.2 \times 10^{-16}$), distance of group centroid to stimulus (LMM: Estimate = 0.022 ± 0.0034; Kenward-Roger F-test: $F_{1,1965}$ = 42.3, $P = 9.7 \times 10^{-11}$), an interaction between bearing of the group heading to the stimulus and polarization (LMM: Estimate = 0.025 ± 0.0029, $F_{1,1963}$ = 69.5, $P < 2.2 \times 10^{-16}$), and cumulative days of testing (LMM: Estimate = -0.019 ± 0.0040; Kenward-Roger F-test: $F_{1,1932}$ = 23.1, $P = 1.6 \times 10^{-6}$), with individual identity nested within group identity as a random effect. The predicted latency at the minimum and maximum observed polarizations O*p* = 0.081 and O*p* = 0.997, and the time difference between the predicated arrival latency at O*p* = 0.081 and O*p* = 0.997, are reported for each order of arrival (also see Fig. 3). For the first fish to arrive at the stimulus, the predicted arrival latency is 0.92 s faster in a disoredered swarm-like group (minimum polarization) compared to a group with maximum polarization. By the fifth fish to arrive at the stimulus the predicted arrival latency is 2.58 s slower in a swarm-like group compared to a group with maximum polarization.

	Predicted latency to arrive at the stimulus (s)		
Arrival Order	Op = 0.081	Op = 0.997	Time difference (s)
1	3.26	4.18	-0.92
2	4.29	4.78	-0.49
3	5.64	5.48	0.16
4	7.42	6.27	1.15
5	9.76	7.18	2.58
6	12.84	8.22	4.62
7	16.90	9.41	7.49
8	22.22	10.77	11.45

Supplementary Table 7 | Model predictions for the effect of the interaction between cumulative days of testing and order of arrival at the stimulus on the latency to arrive at the stimulus. Predictions are generated from the model of latency to arrive at the stimulus specificied in Supplementary Table 6 with the addition of an interaction effect between cumulative days of testing and arrival order at the stimulus (R^2 conditional = 0.67). The predicted latency on day 1 of testing and day 12 of testing, and the time difference between the two, are reported for each order of arrival. All other terms were held at their mean value for the generation of the predictions. The predicted latency to arrive at the stimulus decreases for the 1st to 4th fish and increases for the 5th to 8th fish to arrive at the stimulus over the course of the experiment.

	Predicted latency to arrive at the stimulus (s)		
Arrival Order	Day 1	Day 12	Time difference (s)
1	4.35	2.85	1.50
2	5.08	3.74	1.34
3	5.93	4.90	1.03
4	6.92	6.42	0.50
5	8.08	8.40	-0.32
6	9.43	11.01	-1.58
7	11.01	14.43	-3.42
8	12.85	18.90	-6.05

Supplementary Table 8 | Model predictions for the effect of the interaction between group polarization (O*p*) and order of arrival at the stimulus on the latency to arrive at the stimulus including all observations of fish to arrive at the stimulus within 20 seconds of presentation (n = 2091). Predictions are generated from a model of latency to arrive at the stimulus (no data outliers excluded, R^2 conditional = 0.59) with a highly significant interaction between group polarization and arrival order (LMM: Estimate = -0.024 ± 0.0035, Kenward-Roger F-test: $F_{1,2048} = 48.0$, $P < 2.2 \times 10^{-16}$), and controlling for the significant effects of the bearing of the group heading to the stimulus (LMM: Estimate = 0.091 ± 0.0039; Kenward-Roger F-test: $F_{1,2015} = 490.5$, $P < 2.2 \times 10^{-16}$), distance of group centroid to stimulus (LMM: Estimate = 0.015 ± 0.0040; Kenward-Roger F-test: $F_{1,2016} = 25.1$, $P = 9.6 \times 10^{-11}$), an interaction between bearing of the group heading to the stimulus and polarization (LMM: Estimate = 0.021 ± 0.0033, $F_{1,1839} = 41.65$, $P < 2.2 \times 10^{-16}$), and cumulative days of testing (LMM: Estimate = -0.011 ± 0.0044; Kenward-Roger F-test: $F_{1,2014} = 41.4$, $P = 1.5 \times 10^{-6}$), with individual identity nested within group identity as a random effect. The predicted latency at the minimum and maximum observed polarizations O*p* = 0.081 and O*p* = 0.997, and the time difference between the predicated arrival latency at O*p* = 0.081 and O*p* = 0.997, are reported for each order of arrival.

	Predicted latency to arrive at the stimulus (s)		
Arrival Order	Op = 0.081	Op = 0.997	Time difference (s)
1	3.32	4.10	-0.78
2	4.36	4.71	-0.35
3	5.73	5.41	0.32
4	7.53	6.22	1.31
5	9.90	7.14	2.76
6	13.01	8.20	4.81
7	17.10	9.42	7.68
8	22.48	10.83	11.65

Supplementary Table 9 | Spearman's rank correlations among seven individual-level parameters tested for their effects on the likelihood of an individual being the first responder. Except for body length and proportion of time on the convex hull edge, raw data are median values of each parameter per fish taken from the 0.5 s prior to each stimulus presentation divided by the mean median values of all eight fish in a group. Relative body length and relative proportion of time on the convex hull edge were calculated by dividing by the group mean body length and the group mean proportion of frames on the convex hull of the group, respectively.

	Relative distance to stimulus	Relative bearing to stimulus	Relative proportion of time on convex hull edge	Relative distance to centroid	Relative body length	Relative visual occlusion
Relative speed	0.00	-0.11	0.05	0.03	-0.03	-0.04
Relative distance to stimulus		-0.26	0.02	-0.17	0.01	0.12
Relative bearing to stimulus			-0.02	0.07	0.00	-0.07
Relative proportion of time on convex hull edge				0.63	0.02	-0.53
Relative distance to centroid					0.03	-0.61
Relative body length						-0.06

Supplementary Table 10 | Standardized model averaged coefficient estimates from candidate negative binomial GLMMs to examine the predictors for the response latency of first responders from models excluding group centroid speed. All parameters except for cumulative days of testing are the median values taken from the 13 frames (0.5 sec) prior to each stimulus presentation. Standardized model averaged coefficient estimates (based on the 95% candidate set of models) are reported for each parameter with the upper and lower 95% confidence limits in brackets. The relative importance (RI) of each parameter is the sum of the Akaike weights over the models in which that parameter appears (based on the complete candidate set of models including all possible combinations of main effect terms).

Term	Estimate ($CI_{95\%}$)	RI
Bearing of group heading to stimulus	0.28 (0.209, 0.359)	1.00
Convex hull area	-0.05 (-0.064, 0.041)	0.37
Distance of group centroid to stimulus	0.06 (-0.048, 0.112)	0.58
Polarization	0.11 (0.040, 0.190)	0.97
Cumulative days of testing	-0.29 (-0.372, -0.207)	1.00

Supplementary References

1 Inglis, R. I. & Lazarus, J. Vigilance and flock size in Brent geese: The edge effect. *Z Tierpsychol* **57**, 193-200 (1981).

2 Strandburg-Peshkin, A. *et al.* Visual sensory networks and effective information transfer in animal groups. *Curr. Biol.* **23**, R709-R711 (2013).

3 Nagy, N., Akos, Z. Biro, D. & Vicsek, T. Hierarchical group dynamics in pigeon flocks. *Nature* **464**, 890-893 (2010).

4 Couzin, I. D., Krause, J., James, R., Ruxton, G. D. & Franks, N. R. Collective memory and spatial sorting in animal groups. *J. Theor. Biol.* **218**, 1-11 (2002).

5 Tunstrøm, K. *et al.* Collective states, multistability and transitional behavior in schooling fish. *PLoS Comput. Biol.* **9**, e1002915 (2013).

6 Hunter, J. R. Swimming and feeding behaviour of larval anchovy *Engraulis mordax Fishery Bulletin* **70**, 821-838 (1972).

7 Pita, D., Moore, B. A., Tyrrell, L. P. & Fernandez-Juricic, E. Vision in two cyprinid fish: implications for collective behaviour. *PeerJ* **3**, e1113 (2015).

8 Pérez-Escudero, A., Vicente-Page, J., Hinz, R. C., Arganda, S. & de Polavieja, G. G. idTracker: tracking individuals in a group by automatic identification of unmarked animals. *Nat. Methods* **11**, 743-748 (2014).
