# Supplementary material for: Information can explain the dynamics of group order in animal collective behaviour
Source: Nat Commun. 2020 Jun 1;11:2737. doi: 10.1038/s41467-020-16578-x (PMC7264142; doi:10.1038/s41467-020-16578-x)
Supplement: Supplementary file 2 — Reporting Summary [file 41467_2020_16578_MOESM2_ESM.pdf]

nature research

Corresponding author(s): Hannah E A MacGregor

Last updated by author(s): April 30, 2020

Reporting Summary

Nature Research wishes to improve the reproducibility of the work that we publish. This form provides structure for consistency and transparency in reporting. For further information on Nature Research policies, see Authors & Referees and the Editorial Policy Checklist .

Statistics

For all statistical analyses, confirm that the following items are present in the figure legend, table legend, main text, or Methods section.

n/a	Confirmed	
☐	☒	The exact sample size (n) for each experimental group/condition, given as a discrete number and unit of measurement
☐	☒	A statement on whether measurements were taken from distinct samples or whether the same sample was measured repeatedly
☐	☒	The statistical test(s) used AND whether they are one- or two-sided<br>*Only common tests should be described solely by name; describe more complex techniques in the Methods section.*
☐	☒	A description of all covariates tested
☐	☒	A description of any assumptions or corrections, such as tests of normality and adjustment for multiple comparisons
☐	☒	A full description of the statistical parameters including central tendency (e.g. means) or other basic estimates (e.g. regression coefficient) AND variation (e.g. standard deviation) or associated estimates of uncertainty (e.g. confidence intervals)
☐	☒	For null hypothesis testing, the test statistic (e.g. F, t, r) with confidence intervals, effect sizes, degrees of freedom and P value noted<br>*Give P values as exact values whenever suitable.*
☒	☐	For Bayesian analysis, information on the choice of priors and Markov chain Monte Carlo settings
☒	☐	For hierarchical and complex designs, identification of the appropriate level for tests and full reporting of outcomes
☐	☒	Estimates of effect sizes (e.g. Cohen's d, Pearson's r), indicating how they were calculated

Our web collection on statistics for biologists contains articles on many of the points above.

Software and code

Policy information about availability of computer code

Data collection	Video files were converted using HandBrake version 1.2.0 (https://handbrake.fr/). idTracker version 2.1 (Pérez-Escudero et al. 2014) was used to extract the trajectory data of the fish. ImageJ version 1.52 (https://imagej.nih.gov/ij/download.html) was used to measure standard body lengths from still images.
Data analysis	R statistical software version 3.4.3 (R Development Core Team) was used to carry out linear/generalized linear mixed modeling. MATLAB version 9.5 (R2018b, MathWorks) was used to estimate the visual fields of the fish and visual occlusion.

For manuscripts utilizing custom algorithms or software that are central to the research but not yet described in published literature, software must be made available to editors/reviewers. We strongly encourage code deposition in a community repository (e.g. GitHub). See the Nature Research guidelines for submitting code & software for further information.

Data

Policy information about availability of data

All manuscripts must include a data availability statement. This statement should provide the following information, where applicable:

- Accession codes, unique identifiers, or web links for publicly available datasets
- A list of figures that have associated raw data
- A description of any restrictions on data availability

The data that support the findings of this study are available as Supplementary Data Files 1-5.

Field-specific reporting

Please select the one below that is the best fit for your research. If you are not sure, read the appropriate sections before making your selection.

Life sciences study design

All studies must disclose on these points even when the disclosure is negative.

Sample size	No sample size calculation was performed. Sample sizes were determined by the numbers of fish (n = 96) and group size (n = 8 individuals) deemed appropriate for the study considering natural group sizes in sticklebacks, an expected positive effect of group size on the efficiency of social information acquisition, and the 3R's principals for animal research. Sample sizes were appropriate to the number of explanatory terms in the models and repeated measures.
Data exclusions	Pre-established data exclusion occurred: where the predetermined minimum distance between the fish and the stimulus at presentation was not maintained, in instances where fish required replacement within 4 days of the experiment start to maintain consistency of individuals across the experiment, and where the time of first response to a stimulus could not be quantitatively detected. To meet statistical modeling assumptions, we excluded multivariate outlier data points in one analysis based on an established methodology (Kutner et al. 2005); results from this analysis without data exclusion are reported in the supplementary information. All data exclusions and their rationale are reported.
Replication	Experimental replication occurred at the level of the group (n = 12), trials per group (mean = 6 per group, 77 trials total) and presentations within a trial (n = 6). In 17 presentations, a fish appeared on the same side of the arena as the selected stimulus position during the presentation of the stimulus and therefore the replication was deemed unsuccessful. In these cases, the stimulus presentation was repeated after three minutes as reported.
Randomization	Fish were allocated to groups using a complete random block design as reported.
Blinding	Investigators were blinded to the behaviour of the group when the stimulus was presentation to avoid unconscious bias. Collection of trajectory data was automated using idTracker so blinding was not applicable.

Reporting for specific materials, systems and methods

We require information from authors about some types of materials, experimental systems and methods used in many studies. Here, indicate whether each material, system or method listed is relevant to your study. If you are not sure if a list item applies to your research, read the appropriate section before selecting a response.

Materials & experimental systems

n/a	Involved in the study	
☒	☐	Antibodies
☒	☐	Eukaryotic cell lines
☒	☐	Palaeontology
☐	☒	Animals and other organisms
☒	☐	Human research participants
☒	☐	Clinical data

Methods

n/a	Involved in the study	
☒	☐	ChIP-seq
☒	☐	Flow cytometry
☒	☐	MRI-based neuroimaging

Animals and other organisms

Policy information about studies involving animals; ARRIVE guidelines recommended for reporting animal research

Laboratory animals	N/A
Wild animals	Wild-caught fish were captured using hand nets from the river Cary, Somerset and transported in fish bags to specialist facilities at the University of Bristol. After the experiment the fish were kept and re-used in other behavioural experiments.
Field-collected samples	Juvenile three-spined sticklebacks (Gasterosteus aculeatus) were wild-caught and transported to the environmentally controlled fish laboratory at the University of Bristol. The fish were housed in glass tanks (70 cm (L) × 45 cm (W) × 37.5 cm (H)) of approximately 50 individuals for 10 months before testing and were fed daily with brine shrimp or defrosted frozen bloodworms (Chironomid sp. larvae). During the experiment individuals were housed in their groups in smaller glass tanks (70 (L) × 25 (W) × 37.5 (H) cm). Each holding tank was enriched with a horizontal piece of PVC tubing and an artificial plant. Photoperiod was on 11:13 h light:dark cycle and ambient temperature was maintained at 16°C to prevent the fish from entering reproductive condition. The fish were released at a permitted location following the study.
Ethics oversight	All procedures regarding the use of animals in research followed United Kingdom guidelines and were approved by the University of Bristol Ethical Review Group (UIN UB/17/060).

Note that full information on the approval of the study protocol must also be provided in the manuscript.
