# Supplementary material for: Information can explain the dynamics of group order in animal collective behaviour
Source: Nat Commun. 2020 Jun 1;11:2737. doi: 10.1038/s41467-020-16578-x (PMC7264142; doi:10.1038/s41467-020-16578-x)
Supplement: Supplementary file 3 — Description of Additional Supplementary Files [file 41467_2020_16578_MOESM3_ESM.pdf]

Description of Additional Supplementary Files

File Name: Supplementary Data 1
Description: Individual-level variables used in the analyses of the likelihood to respond first to the stimulus.

File Name: Supplementary Data 2
Description: Group-level variables used in the analyses of the latency to first respond to the stimulus and collective visual field data.

File Name: Supplementary Data 3
Description: Variables used in the analyses of the latency to arrive at the stimulus.

File Name: Supplementary Data 4
Description: Individual-level variables used in the analysis of the heading angle to nearest neighbours.

File Name: Supplementary Data 5
Description: Data on food item consumption.

File Name: Supplementary Movie 1
Description: Examples of responses to the stimulus from groups with low levels of collective order and high levels of collective order.

File Name: Supplementary Movie 2
Description: Illustration of the effects of different levels of collective order for the proportion of the arena within the visual range of a group based on a binocular (30°) field of view.
